# Sleepiness, fatigue, anxiety and depression in Chronic Obstructive Pulmonary Disease and Obstructive Sleep Apnea – Overlap – Syndrome, before and after continuous positive airways pressure therapy

**DOI:** 10.1371/journal.pone.0197342

**Published:** 2018-06-11

**Authors:** Nicholas-Tiberio Economou, Ioannis Ilias, Lemonia Velentza, Yiannis Papachatzakis, Paul Zarogoulidis, Anastasios Kallianos, Georgia Trakada

**Affiliations:** 1 Division of Pulmonology, Department of Clinical Therapeutics, National and Kapodistrian University of Athens School of Medicine, Alexandra Hospital, Athens, Greece; 2 Endocrine Unit, Elena Venizelou Hospital, Athens, Greece; 3 Pulmonary Department-Oncology Unit, "G. Papanikolaou" General Hospital, Aristotle University of Thessaloniki, Thessaloniki, Greece; University of Rome Tor Vergata, ITALY

## Abstract

Patients with Chronic Obstructive Pulmonary Disease (COPD) and / or Obstructive Sleep Apnea (OSA) often complain about sleepiness, fatigue, anxiety and depression. However, common screening questionnaires, like Epworth Sleepiness Scale (ESS), Fatigue Severity Scale (FSS) and Hospital Anxiety and Depression Scale (HADS) have not been previous evaluated in patients with overlap–coexisting COPD and OSA–syndrome versus patients with OSA alone. Our study compared ESS, FSS and HADS between patients with overlap syndrome and patients with OSA, before and after treatment with Continuous Positive Airways Pressure (CPAP). We examined 38 patients with coexisting COPD and OSA versus 38 patients with OSA-only and 28 subjects without respiratory disease, serving as controls. All patients underwent pulmonary function tests (PFTs), oximetry and overnight polysomnography and completed the questionnaires, before and after 3 months of CPAP therapy. The two patient groups did not differ significantly in terms of age, Body Mass Index (BMI), neck, waist and hip circumferences, and arterial blood pressure values. They also had similar comorbidities. They differed significantly, as expected, in PFTs (Forced Vital Capacity–FVC, 2.53±0.73 vs 3.08±0.85 lt, p = 0.005, Forced Expiratory Volume in 1sec–FEV1, 1.78±0.53 vs 2.60±0.73 lt/min, p<0.001) and in daytime oximetry (94.75±2.37 vs 96.13±1.56%, p = 0.007). ESS, HADS–Anxiety and HADS–Depression scores did not differ statistically significant between these two groups, whereas overlap syndrome patients expressed significantly more fatigue (FSS) than OSA-only patients, a finding that persisted even after 3 months of CPAP therapy.

We conclude that sleepiness, anxiety and depression were similar in both groups, whereas fatigue was more prominent in patients with overlap syndrome than in sleep apneic patients and did not ameliorate after treatment.

## Introduction

The ‘overlap syndrome’ was first described by Flenley as the coexistence of COPD and OSA [1]. COPD is defined as a preventable and treatable, systemic disease, characterized by air-flow limitation that is not fully reversible [2]. Spirometric criteria (Forced Expiratory Volume in 1 second [FEV1] and ratio of FEV1 to Forced Vital Capacity [FVC] after bronchodilation < 0.70) are used to confirm the diagnosis and to assess the severity of the disease [2]. OSA is defined as multiple substantial decreases (hypopneas) or complete cessations (apnoeas) of airflow during sleep, despite increased effort to breathe, due to collapse of the upper airway, which lead to repetitive oxygen desaturations, brief arousals and sleep fragmentation [3]. The prevalence of overlap syndrome in adults aged 40 years and over is estimated about 0.5%–1% [4]. Although COPD and OSA are both highly prevalent diseases, it is unclear if each disorder predisposes to a higher incidence of the other [4].

Both diseases are characterized by severe clinical symptoms and are associated with significant morbidity and mortality. Thus, one could anticipate that patients with overlap syndrome may have a worse symptomatology and prognosis than patients with only one of either disease.

Patients with COPD often report poor sleep quality, fatigue, daytime sleepiness and impaired quality of life [5, 6]. Furthermore, they often suffer from anxiety and / or depression [7]. Patients with OSA usually complain about unrefreshing sleep, excessive daytime sleepiness and fatigue [8]. Moreover, a strong association exists between anxiety and depression and sleep apnea [9]. There are no specific symptoms for patients with overlap syndrome. Usually a patient known to have either OSA or COPD is evaluated by a primary care physician, pulmonologist, or sleep specialist. OSA patients with a smoking history and daytime respiratory symptoms, hypoxemia or hypercapnia should be referred for PFTs. COPD patients with pulmonary hypertension, daytime hypoventilation and obesity should be referred for polysomnography. COPD diagnosis is simple and inexpensive (2), whereas OSA diagnosis requires an overnight polysomnography, a time-consuming and expensive test of limited availability [10].

Recently, Mermigkis et al [11] showed that although overlap syndrome patients had significantly worse quality of life, when compared to COPD-only controls, they did not report excessive daytime sleepiness or elevated Epworth sleepiness score, which highlights the difficulties with clinical diagnosis and screening. In another study, ESS did not accurately predict OSA in the group of patients with COPD [12]. Furthermore, there is limited evidence about the impact of CPAP therapy on symptomatology. Common screening questionnaires for sleepiness, fatigue, depression and anxiety have not been previously evaluated in overlap syndrome patients versus OSA-only patients.

Our study aimed to compare ESS, FSS and HADS questionnaires between overlap syndrome patients and OSA-only controls, before and after three months of CPAP treatment, in order to know the impact of sleepiness, fatigue, depression and anxiety in this population and to point out the role of therapy in symptoms.

## Patients and methods

Thirty eight (38) patients (27 men, 11 women) with newly-diagnosed COPD and coexisting OSA–overlap syndrome–and 38 newly-diagnosed, case-control, OSA-only patients were selected for the present study from our outpatient clinic in the Division of Pulmonology, Department of Clinical Therapeutics of the National and Kapodistrian University of Athens School of Medicine, at Alexandra Hospital of Athens, between 1^st^ January 2014 to 30^th^ June 2016. A sample of patients that presented to the respiratory outpatient clinic with various complaints and who were subsequently found not to suffer from COPD and/or OSAS (after a thorough diagnostic work-up), were included as a control group.

Our clinic is a general pulmonary division into an internal clinic that deals with all pulmonary diseases. Written informed consent was obtained from all individual participants included in the study and the study protocol was approved by the Ethical Committee of the “Alexandra” University hospital, before the initiation of the study. (approval number: 261/07.05.2013) The recruitment and the reassessment of the patients was done from one pulmonologist (Dr G.T.).

COPD was diagnosed by history, physical examination, and standard pulmonary function tests according to GOLD criteria [2]. Any patient who had dyspnea, chronic cough or sputum production, and / or history of exposure to risk factors for the disease underwent spirometry and static lung volumes measurement to confirm the presence of persistent airflow limitation. When COPD was stable, under optimal bronchodilation therapy and optimal therapy of comorbidities, patients were eligible to participate in the study.

OSA was diagnosed as an Apnea-Hypopnea Index (AHI) of ≥5 events per hour of sleep with associated symptoms or comorbidities or an AHI of ≥15, regardless of associated symptoms or comorbidities, according to American Academy of Sleep Medicine (AASM) diagnostic criteria [13]. COPD patients that reported poor sleep quality and / or unrefreshing sleep underwent standard full night polysomnography.

Exclusion criteria were oxygen supplementation, other lung diseases, sleep disorders other than OSA (e.g., narcolepsy, periodic limb movements, central sleep apnea [central apnea index ≥5/h]), active or unstable cardiovascular diseases, non-controlled arterial hypertension, severe dementia, severe untreated psychiatric conditions and unwilling, undisciplined patient who do not comply with medical recommendations.

At baseline, each patient underwent clinical examination, oximetry (model 8800, Nonin Medical, Inc., Plymouth, MN), and FFTs (Master screen Diffusion, Jaeger, Germany): spirometry (pre/post bronchodilation) and whole-body plethysmography determination of static lung volumes. BMI, neck, waist and hip circumferences, and arterial blood pressure values were also measured.

All patients answered ESS, FSS and HADS. The ESS is a self-report questionnaire that evaluates the tendency to fall asleep in eight daily situations. The ESS score ranges from 0 to 24, and a score ≥10 indicates excessive daytime sleepiness [14]. FSS is a questionnaire with nine questions estimating the fatigue severity in different situations during the past week. Grading ranges from 1 (strong disagreement) to 7 (strong agreement) where the final score is the mean value of the nine items, and a score ≥4 is interpreted as fatigue [15]. HADS is also a self-report, 14-item depression and anxiety screening instrument assessing the severity of symptoms in medically ill patients, while excluding somatic symptoms potentially attributable to comorbid medical conditions [16]. Each question is scored from 0–3, with a total score up to 21 for each subscale.

A standard full night polysomnography (PSG) was performed in each patient the same night of the initial evaluation (Alice, Respironics, Murraysville, PA). Sleep records were scored according to standard criteria and manually revised by an expert [17].

Apnea was defined as a drop in the peak signal excursion by ≥ 90% of pre-event baseline using an oronasal thermal sensor (diagnostic study), positive airway pressure device flow (titration study), or an alternative apnea sensor, when the duration of the ≥ 90% drop in sensor signal was ≥ 10 seconds. Hypopnea was defined as the breathing reduction of ≥30% of pre-event baseline that exceeded 10 sec and was associated with ≥3% oxygen desaturation from pre-event baseline or with an arousal. Arousal was defined as an abrupt shift in electroencephalograph (EEG) frequency including alpha, theta and / or frequencies greater than 16Hz (but not spindles) that lasts at least 3sec, with at least 10sec of stable sleep preceding the change and increased mylohyoid electromyographic (EMG) signal of >1sec during REM sleep. AHI was defined as the total number of apneas and hypopneas per hour of electroencephalographic sleep. A second sleep study for manually CPAP titration followed the next night. The optimum CPAP pressure was defined as the pressure value that abolished all respiratory events, arousals, and desaturation episodes. CPAP adherence was estimated by dividing total recorded hours in the device timer by the number of nights of the use between treatment initiation and follow-up examination after a three months period. Adherence was considered as acceptable, when CPAP usage was >4h per night [18]. At the end of the 3 months period of therapy, the patients were reassessed using the same protocol.

Normality of the parameters’ distribution was assessed with the Wilks-Shapiro test. The values of parameters were expressed as mean and standard deviation (M±SD) or as quartiles (if not normally distributed). One-way analysis of variance (ANOVA) was conducted to compare age, body mass index, lung function tests and PSG parameters over the three groups (overlap, OSAS and control group) before CPAP therapy. The parameters which were not normally distributed were compared with the Kruskall Wallis test. One-way analysis of covariance (ANCOVA) was conducted to compare the questionnaires’ scores (ESS, FSS, HADS-A and HADS-D) over the three groups (overlap, OSAS and control group) before CPAP therapy whilst controlling for age. Levene’s test and normality checks were carried out and the assumptions met. Univariate and multivariate analyses were used to determine possible correlations between anthropometric data and questionnaires’ scores and AHI. Student’s t-test was used for the comparison of the questionnaires’ values after three months of CPAP therapy. A p value lower than 0.05 was considered as being significant.

## Results

**Fig 1:**
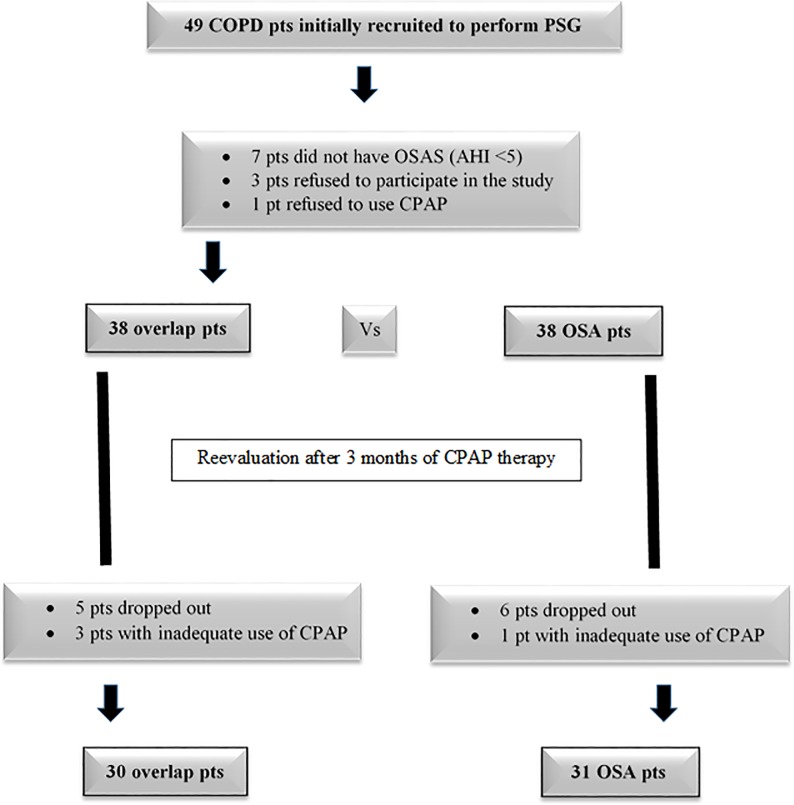
Study protocol.

**Fig 2:**
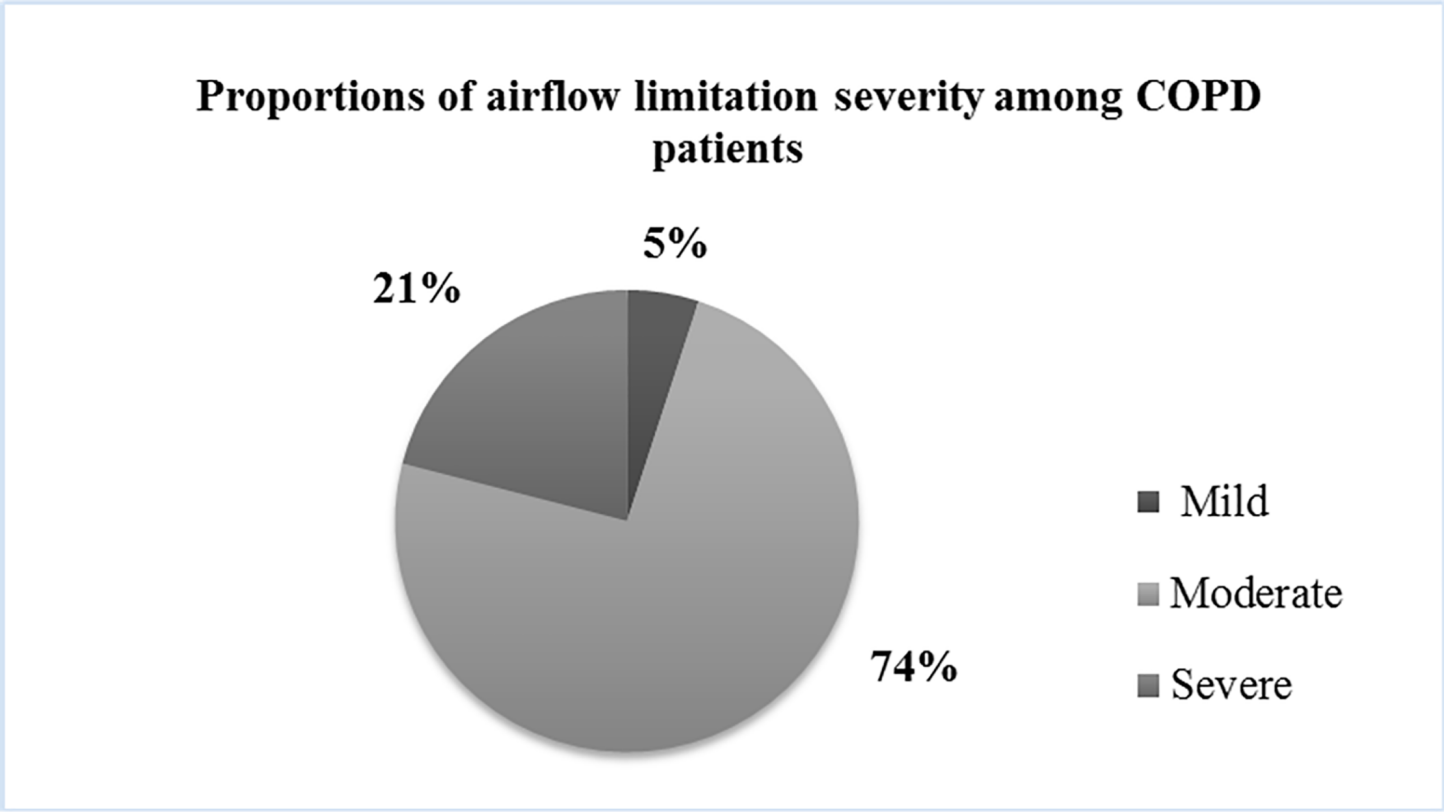
Severity of airflow limitation in COPD patients.

A total of 49 COPD patients were recruited during our study period. Three patients refuged to perform the diagnostic sleep study, 7 patients had and AHI <5 and 1 patient refused CPAP titration and were excluded from the study. The remaining 38 newly-diagnosed patients, 27 men and 11 women, with overlap syndrome were included in the study, as well as 38 case-control patients with OSA-only, with mean age 67 years old “Fig 1”. The baseline characteristics of the study population are presented in Table 1. There was a significant effect of age on group at the p < .05 level [F(2, 102) = 14.60, p = 0.001]. Post hoc comparisons using the Tukey HSD test indicated that age was significantly lower for the control group vs overlap or OSAS group. No differences among groups were noted for BMI. As expected, overlap syndrome patients had significantly lower pulmonary function when compared to OSA-only patients. “Fig 2” presents airflow limitation severity among COPD patients. Three (3) overlap (7.89%) and 9 OSA patients (23.69%) had no other known diseases. The most common coexisting diseases in both groups were hypertension, cardiovascular disease (CVD), diabetes mellitus (DM) and dyslipidemia. No statistically significant difference was observed in the number and the type of comorbidities between the two groups. PSG data are presented in Table 2. Patients had more severe OSA, in terms of AHI vs controls whereas overlap patients had worse meanSaO2 vs controls. OSA-only patients had significantly longer respiratory events and reached a significantly lower minSaO2, when compared to overlap syndrome patients. Both patient groups expressed similar degree of sleepiness (ESS), anxiety (HADS-A) and depression (HADS-D) (Table 3). Almost half of the patients expressed daytime sleepiness (ESS ≥10), whereas only 2 overlap and 1 OSA patients expressed anxiety or depression (HADS ≥11). The overlap syndrome patients were more fatigued than OSA-only patients [FSS: 4.4±1.84 vs 4.37±1.6, F(2,70) = 2.00, p = 0.02], whilst adjusting for age, although a similar percentage of both groups (about 60%) expressed fatigue (FSS ≥4). The control subjects had higher anxiety and marginally higher depression scores than both patient groups [F(2, 70) = 4.95, p = 0.01 and F(2,70) = 2.28, p = 0.06], whilst adjusting for age.

**Table 1:** Baseline characteristics of the study groups (mean±SD).

	Overlap Syndrome (COPD + OSA)	OSA	Control group
Age (years)	66.9±8.6	67.0±9.2	52.9±11.8+
BMI (kgr/m^2^)	35.4±8.6	34.7±7.9	32.2±5.3
Neck circumference (cm)	42.9±4.5	42.0±4.2	38.2±3.9+
Waist circumference (cm)	118.9±13.1	117.1±14.1	106.8±12.5+
Hip circumference (cm)	112.5±12.7	115.1±11.3	112.5±10.4
Systolic Arterial Blood Pressure (mmHg)	127.5±12.3	125.9±17.2	125.8±12.4
Diastolic Arterial Blood Pressure (mmHg)	79.3±8.8	81.7±8.3	83.2±11.6
FVC (L)	2.53±0.73*	3.08±0.85**	3.58±1.09
FEV1 (L)	1.78±0.52*,**	2.6±0.73**	2.98±0.90
FEV1/FVC	0.70±0.86*	0.83±0.51	0.83±0.47
PEF (L/min)	4.76±1.73*, #	6.22±2.12	7.22±2.54
SaO2 (%)	94.7±2.4*, #	96.1±1.6	96.9±1.6

* p<0.01 Overlap vs OSA group

+ p<0.01 Overlap & OSA vs control group

** p<0.001 OSA vs control group

# p<0.01 Overlap vs control group

Abbreviations: BMI: Body Mass Index, FVC: Forced Vital Capacity, FEV1: Forced Expiratory Volume in 1sec, PEF: Peak Expiratory Flow Rate, SaO2: Oxyhemoglobin Saturation.

**Table 2:** Sleep characteristics of the three groups (mean±SD or 1^st^ quartile/median/3^rd^ quartile).

	Overlap Syndrome (COPD + OSA)	OSA	Control group
Total Recording Time (min)	307.73±109.11+	301.75±116.1+	389.54±43.68
TST (min)	241.68±92.37+	229.73±104.73+	312.22±73.56
SE (%)	77.17±17.95	76.55±18.86	80.05±16.94
Sleep onset (min)	5.87/11.70/21.80+	4.50/12.25/26.00+	9.10/22.90/46.50
REM sleep onset (min)	152.3±100.09	152.04±93.83	176.79±62.36
WASO (min)	5.50/30.40/90.50	4.65/32.90/57.50	7.22/25.50/51.00
N1 (min)	7.00/18.00/46.50	5.00/13.20/46.50	5.75/8.50/16.90
N2 (min)	74.50/141.00/204.50	76.20/117.40/160.00	115.00/154.00/232.07
N3 (min)	00.00/24.75/56.00+	00.00/16.50/80.00+	37.75/68.00/119.50
REM (min)	00.00/14.00/36.00	00.00/12.00/48.00	18.25/32.75/59.10
Apneas + Hypopneas (n)	93.00/150.00/262.00+	99.00/137.00/203.00+	15.50/25.50/41.00
Obstructive Apneas (n)	10.00/43.50/121.00	19.00/61.00/116.00	0.00/1.50/7.00+
Central Apneas (n)	0.00/2.00/14.00	0.00/2.00/19.00	0.00/1.00/5.50+
Hypopneas (n)	30.00/64.00/150.00	33.00/63.00/86.00	5.00/14.50/29.00+
AHI	50.68±31.71	50.16±26.91	5.95±3.46+
AHI nREM	54.96±32.74	48.56±28.49	5.52±2.74+
AHI REM	47.1±30.71	43.43±20.22	10.74±8.98+
AI	6.87/10.40/25.27	9.57/14.30/30.85	20.30/20.95/22.40
Μean duration of events (sec)	21.40±5.62#	21.97±5.98	19.77±4.81
Μax duration of events (sec)	53.35±18.01#	57.26±23.13	41.13±20.16+
Μean SaO2 (%)	91.15±2.76#	92.4±2.8	93.21±2.16
Μin SaO2 (%)	80.08±6.87+	79.11±8.58+,#	86.75±4.03
SaO2 <90% (%)	0.55/6.80/38.40	1.00/8.10/21.97	0.10/0.40/1.65+

+ p<0.01 Overlap & OSA vs control group

# p<0.01 Overlap vs control group

Abbreviations: TST: Total Sleep Time, SE: Sleep Efficiency, WASO: Wake after Sleep Onset, N1: Stage 1, N2: Stage 2, N3: Stage 3–4, REM: Rapid Eye Movement, AHI: Apnea-Hypopnea Index, AI: Arousal Index, SaO2: Oxyhemoglobin Saturation.

**Table 3:** Questionnaires’ results.

	Overlap Syndrome (COPD + OSA)	OSA	Control group
ESS t = 0	10.97±4.88	10.08±4.95	7.35±4.72+
ESS t = +3 months CPAP	7.25±5.31	9.11±4.94	NA
FSS t = 0	4.40±1.84	4.37±1.60*	4.33±1.57+
FSS t = +3 months	3.48±1.98	3.33±1.41*,#	NA
HADS-A t = 0 CPAP	4.76±4.10+	4.14±2.88+	7.77±4.75
HADS-A t = +3 months CPAP	3.33±3.04*	3.86±3.89	NA
HADS-D t = 0	5.48±3.17	4.43±2.66+	7.11±4.24
HADS-D t = +3 months CPAP	2.89±2.62	5.14±3.18	NA

NA: not applicable

*p<0.05 Overlap vs OSA group

+p<0.01 Overlap and/or OSA vs control group

#p<0.05 before vs after CPAP

**Fig 3:**
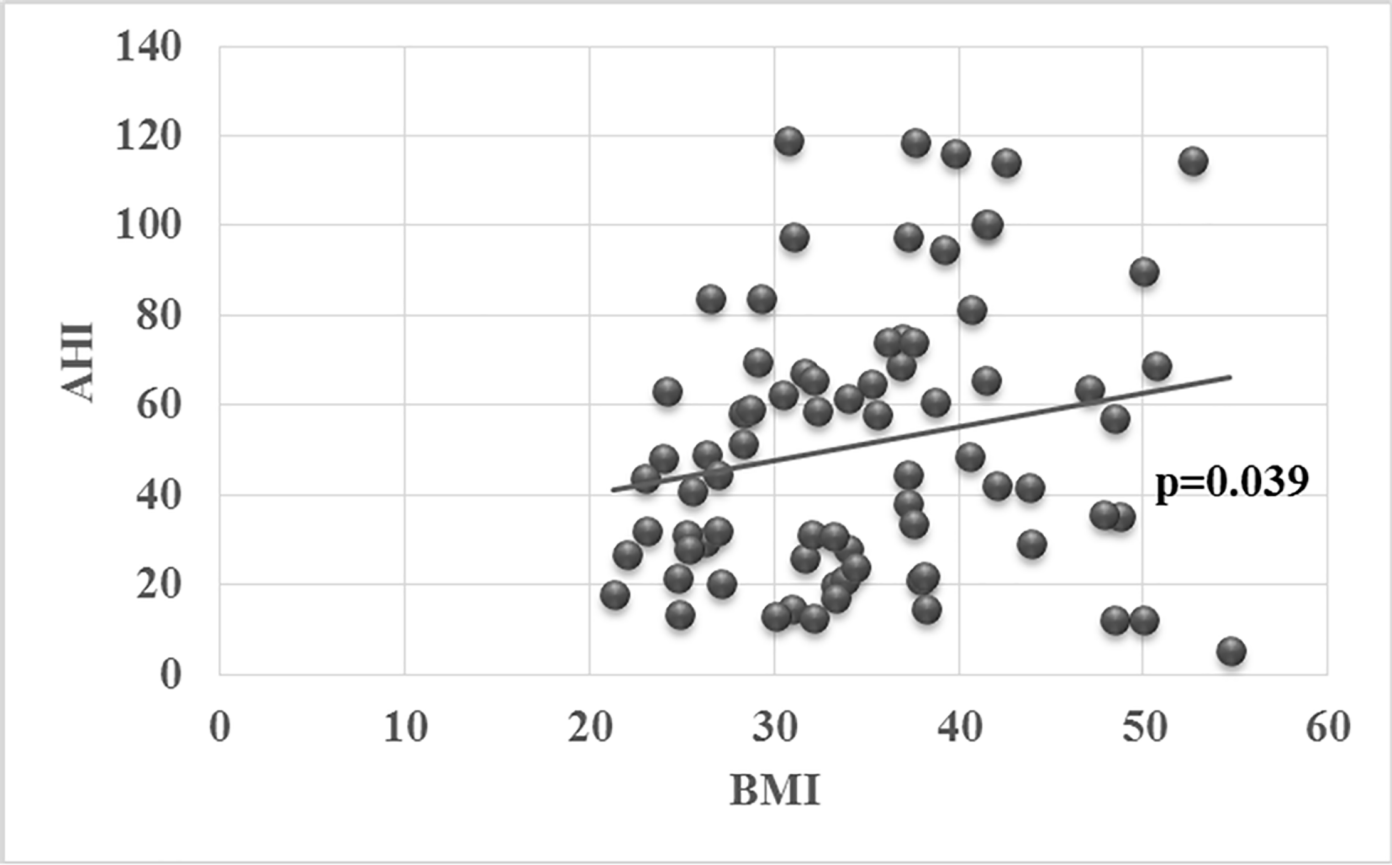
Correlation between BMI and AHI.

In the univariate analysis none of the scores in questionnaires correlated with AHI (ESS: p = 0.680, FSS: p = 0.212, HADS-A: p = 0.240, HADS-D: p = 0.577). Furthermore, in the multivariate analysis only male gender and BMI correlated positively with AHI (p = 0.046, and p = 0.039, respectively) “Fig 3”, whereas age and degree of airways obstruction—as expressed by FEV_1_—did not (p = 0.457, and p = 0.761, respectively). During the reassessment, 5 overlap and 6 OSA patients dropped out. Furthermore, 3 overlap and 1 OSA patients were excluded because of inadequate use of CPAP (<4 hours per night) “Fig 1”. The residual AHI during CPAP use, as measured by the CPAP machine, averaged 3.03 events per sleep in overlap syndrome patients and 3.04 in OSA-only patients, indicating good control of obstructive sleep apnea with CPAP. Although both groups had good adherence to treatment (≥4 hours per night) during follow-up, patients with OSA and coexisting COPD used significantly less hours CPAP than OSA-only patients. Moreover, FEV1 significantly increased in overlap syndrome patients after therapy, when compared to FEV1 before therapy (1.78±0.53 vs 1.93±0.54lt, p = 0.011, respectively). Both patient groups expressed again similar degree of sleepiness (ESS) and depression (HADS-D) (Table 4). Overlap syndrome patients remained more fatigued than OSA-only patients (FSS), whereas OSA-only patients became significantly more anxious than overlap syndrome patients (HADS-A) (Table 4). All three questionnaires did not change statistically significant in overlap syndrome patients, before and after 3 months of CPAP therapy. Only FSS significantly decreased in OSA-only patients after CPAP therapy, whereas the other questionnaires did not show significant changes. There were no significant correlations of ESS or FSS neither with HADS-A, nor with HADS-D. ESS showed a marginal negative correlation (r = -0.663, p = 0.051) with duration of CPAP use in OSAS only patients, whereas no statistically significant difference was observed between FSS and hours of CPAP use between the two groups (p = 0.5257 in overlap patients and p = 0.8618 in OSA-only patients). Duration of CPAP use was positively correlated with HADS-D (r = +0.595, p = 0.041) in overlap patients.

**Table 4:** Characteristics of the study groups (mean±SD) after 3 months of CPAP.

	Overlap Syndrome (COPD + OSA)	OSA
Neck circumference (cm)	42.1±4.1	43.1±4.1
Waist circumference (cm)	117.0±13.6	113.1±15.2
Hip circumference (cm)	116.8±13.9	110.9±10.2
Systolic Arterial Blood Pressure (mmHg)	128.2±11.0	128.3±16.6
Diastolic Arterial Blood Pressure (mmHg)	79.6±6.9	77.5±11.2
FVC (L)	2.65±0.76	3.02±0.83
FEV1 (L)	1.98±0.52*	2.51±0.74
FEV1/FVC	0.76±0.08*	0.83±0.07
PEF (L/min)	5.06±1.92	6.44±2.42
SaO2 (%)	96.4±0.6	96.5±1.3

*p<0.05 Overlap vs OSA group

## Discussion

Our study is the first case-control study assessing simultaneously sleepiness, fatigue, anxiety and depression in overlap syndrome patients, based on three practical and well-validated questionnaires—ESS, FSS and HADS—before and after 3 months of CPAP therapy. Although those symptoms are very common in OSA-only or in COPD-only patients, data are inconsistent when the two diseases coexist. Moreover, the role of CPAP therapy on symptomatology is not well validated in these patients.

Sleepiness express a subjective difficulty in maintaining an awake state, and an increase ease of falling asleep when a person is sedentary. The total ESS score gives an estimate of the personal average sleep propensity, across a wide range of activities in the daily life (14). Moderate to severe excessive daytime sleepiness is observed in about 10% of the general population according to a large epidemiological study [19]. In sleep clinics, sleepiness is a cardinal symptom, mainly due to sleep fragmentation, as a result of apnea or due to sleep deprivation, as a result of socioeconomics conditions (work, life style etc.). However, the association between sleepiness and sleep apnea is weak and in the Sleep Heart Health Study, only 16% of those with an AHI > 5 reported sleepiness [20]. Furthermore, although sleepiness is a good predictor for compliance to therapy, a residual degree remains even after correction of sleep apnea with CPAP [21]. In COPD population, 23% of patients complain of excessive daytime sleepiness [22] and poor sleep quality is the third most common symptom, after dyspnea and fatigue [23].

In a recent study, COPD-only patients reported similar degree of sleepiness in ESS, when compared to overlap syndrome patients [11]. In another study, ESS did not accurately predict OSA in the group of patients with COPD [12]. In our study, half of the patients were sleepy (ESS ≥ 10) in the initial evaluation and one third of the patients remained sleepy in the reassessment. However, the mean score did not differ statistically significant between overlap syndrome and OSA-only patients, before and after CPAP therapy. These data suggest that sleepiness is not a symptom that can distinguish the two groups and that CPAP partially reverse the increased propensity for sleep. Probably, poor sleep quality—that characterizes both OSA and COPD—is only one determinant of sleepiness in clinical and population samples, and other factors like age, obesity, diabetes, insulin resistance, physical activity, depression, and endogenous, genetic characteristics may contribute to the expression of this symptom, both in patients and in healthy subjects [19, 24].

Fatigue express tiredness and lack of energy without increased sleep propensity and FSS is the most commonly used fatigue specific questionnaire [15]. Although many disorders, including sleep-wake disorders, can cause fatigue, it is underrecognized, both in research projects and in daytime clinical work. OSA patients complain of fatigue at least equal to sleepiness [25]. In a previous study, fatigue, tiredness, and lack of energy were more often reported when compared to sleepiness (57%, 61%, and 62% vs 47%, respectively) and the one most significant symptom was lack of energy (about 40%) than any other problem, including sleepiness (about 22%) [25]. However, sleep clinics usually evaluate sleepiness and not fatigue. Limited data also exist about the impact of CPAP therapy on fatigue. Tomfohr LM et al [26] showed that 3 weeks of therapeutic CPAP significantly reduced fatigue and increased energy in patients with OSA, especially in those with high levels of fatigue at the beginning. In COPD patients, fatigue is the second most common symptom (47–71% prevalence in different studies) and can be considered as the main extrapulmonary clinical feature of the disease, which impacts significantly on the health-related quality of life of the patients. [23]. Although decreased FEV_1_ is associated with increased severity and impact of fatigue in COPD in one recent study [27], the experience of fatigue in this population seems to be related mainly to dyspnea, depression, and insomnia [6].

No data exist about fatigue in overlap syndrome. In our study, more than half of our patients of both groups complained about fatigue (FSS ≥ 4) in the initial evaluation and overlap syndrome patients expressed significantly more fatigue than OSA-only patients. In the reassessment, half of the overlap patients continued to complain about fatigue versus one out of five of OSA-only patients. Moreover, the symptom remained statistically more severe in overlap patients when compared to OSA-only patients, after CPAP therapy and even though FEV_1_ increased statistically significant. A possible mechanism may be the enhanced inflammation in overlap syndrome compared to OSA-only.

Psychiatric comorbidities, like depression and / or anxiety, are often observed in patients suffering from chronic somatic diseases. HADS is a well-validated and sensitive to change, screening instrument, and a score between 0–7 is considered as normal [16]. In general population, the prevalence of anxiety disorders is 14% and of depressive disorders 7.8% [28]. In OSA, the reported prevalence of depression or anxiety is approximately 30% [29], whereas in COPD is around 50% [30]. These symptoms affect the quality of life and the prognosis of the patients, and are independently associated with poorer adherence to therapy [31].

No previous data also exist about anxiety and depression in overlap syndrome. In our study, percentages of anxiety and depression were lower than previously reported in either disease, OSA-only or COPD-only. However, they were similar with previously reported data in internal medicine outpatients (HADS-A 8.7±4.3, HADS-D 7.3±3.8) and controls (HADS-A 5.5±3.6, HADS-D 4.0±3.1), in Greece [32]. Moreover, in a recent systematic review and meta-analysis [33], positive airway pressure treatment in OSA had a moderate clinical effect on symptoms of depression and anxiety that was mainly mediated by patient expectations and contact with healthcare providers.

Our study has several limitations. One is the relatively restricted number of patients. COPD and OSA are heterogeneous diseases, characterized by different clinical outcomes and prognosis, despite similar airway obstruction or apnea–hypopnea index. CPAP adherence was also assessed from the proposed algorithm of two types of CPAP machines. Furthermore, we did not measure markers of inflammation, like C-reactive protein (CRP) that can contribute to fatigue and / or sleepiness. Finally, we did not record sleep habits (time in bed, naps) and duration that can act as confounding factors in symptoms. Our control subjects had higher anxiety and marginally higher depression scores despite better lung function and better sleep. Possibly this finding reflects a lurking somatoform disorder in some of them, taking into account the fact that their workup results were normal despite their reported complaints.

Our study was the first evaluating sleepiness, fatigue, depression and anxiety in overlap syndrome versus OSA-only, as both clinical symptoms and the role of CPAP treatment in these patients is not well defined. Fatigue was more pronounced than sleepiness, depression or anxiety. Fatigue should be evaluated whenever there is clinical suspicion of overlap syndrome. However, CPAP therapy for 3 months failed to ameliorate this symptom.

## Supporting information

S1 File Population of overlap SAS controls. (DOCX)
