# Supplementary material for: Sleepiness, fatigue, anxiety and depression in Chronic Obstructive Pulmonary Disease and Obstructive Sleep Apnea – Overlap – Syndrome, before and after continuous positive airways pressure therapy
Source: PLoS One. 2018 Jun 11;13(6):e0197342. doi: 10.1371/journal.pone.0197342 (PMC5995394; doi:10.1371/journal.pone.0197342)
Supplement: S1 File — (DOCX) [file pone.0197342.s001.docx]

S1 File

| **SEX** | **GROUP** | **AGE** | **ΒΜΙ** | **NECK** | **WAIST** | **HIP** | **DBP (mmHg)** | **SBP (mmHg)** | **SMOKING (PY)** |
| --- | --- | --- | --- | --- | --- | --- | --- | --- | --- |
| M | 1 | 53 | 39.8 | 51 | 128 | 127 | 60 | 105 | 120 |
| M | 2 | 53 | 40.7 | 44 | 124 | 129 |  |  | 30 |
| M | 1 | 57 | 37.2 | 56 | 142 | 126 | 80 | 140 | 80 |
| M | 2 | 57 | 37.2 | 42 | 128 | 124 | 80 | 120 | 33 |
| M | 1 | 55 | 31.6 | 41 | 119 | 112 | 80 | 120 | 80 |
| M | 2 | 55 | 30.5 | 39 | 112 | 114 | 80 | 120 | 10 |
| M | 1 | 68 | 35.3 | 44 | 128 | 118 | 90 | 140 | 80 |
| M | 2 | 69 | 33.3 | 45 | 117 | 115 | 65 | 115 | 15 |
| F | 1 | 44 | 37.5 | 34 | 127 | 128 | 80 | 120 | 35 |
| F | 2 | 45 | 38.2 | 40 | 123 | 135 |  |  | 0 |
| F | 1 | 54 | 48.7 |  |  |  |  |  | 50 |
| F | 2 | 55 | 50 |  |  |  |  |  | 0 |
| F | 1 | 67 | 39.2 | 41 | 117 | 126 |  |  | 15 |
| F | 2 | 68 | 38.7 |  |  |  |  |  | 0 |
| M | 1 | 77 |  |  |  |  |  |  | 0 |
| M | 2 | 76 | 24 |  |  |  |  |  | 0 |
| M | 1 | 68 | 24.9 |  |  |  |  |  | 0 |
| M | 2 | 68 | 25.6 | 39 | 99 | 101 | 90 | 105 | 0 |
| M | 1 | 80 | 23.1 | 43 | 97 | 94 | 70 | 120 | 0 |
| M | 2 | 77 | 21.3 | 37 | 99 | 102 |  |  | 0 |
| F | 1 | 75 | 50.7 | 44 | 131 | 137 |  |  | 15 |
| F | 2 | 74 | 47.1 | 50 | 135 | 120 | 80 | 140 | 50 |
| M | 1 | 71 | 26.2 | 42 | 108 | 101 | 70 | 110 | 30 |
| M | 2 | 70 | 25.3 | 40 | 105 | 102 | 80 | 120 | 20 |
| M | 1 | 54 | 32.3 | 45 | 109 | 111 |  |  | 45 |
| M | 2 | 53 | 42.1 | 45 | 106 | 112 | 90 | 150 | 0 |
| M | 1 | 57 | 31 | 41 | 113 | 116 | 90 | 140 | 60 |
| M | 2 | 57 | 32.1 | 48 | 121 | 116 | 80 | 120 | 50 |
| M | 1 | 56 | 29.1 | 41 | 115 | 110 | 80 | 140 | 0 |
| M | 2 | 58 | 28.35 | 41 | 111 | 115 |  |  | 20 |
| M | 1 | 69 | 30.1 | 41 | 110 | 110 | 80 | 120 | 50 |
| M | 2 | 69 | 29.3 |  |  |  |  |  | 0 |
| M | 1 | 76 | 26.9 | 42 | 107 | 94 | 85 | 145 | 40 |
| M | 2 | 78 | 28.3 | 45 | 104 | 99 | 70 | 110 | 0 |
| M | 1 | 56 | 41.5 |  |  |  |  |  | 70 |
| M | 2 | 58 | 41.5 | 42 | 139 | 131 | 85 | 140 | 30 |
| M | 1 | 76 | 43.8 | 45 | 143 | 139 | 96 | 130 | 45 |
| M | 2 | 72 | 42.5 |  |  |  |  |  | 50 |
| F | 1 | 60 | 37.2 | 39 | 126 | 119 | 70 | 115 | 30 |
| F | 2 | 59 | 37.9 |  |  |  |  |  | 0 |
| M | 1 | 62 | 23 | 42 | 94 | 103 | 90 | 140 | 20 |
| M | 2 | 61 | 22 | 32 | 92 | 99 | 80 | 120 | 30 |
| M | 1 | 70 | 38.1 | 45 | 125 | 112 | 80 | 140 | 0 |
| M | 2 | 71 | 36.9 | 45 | 127 | 118 | 85 | 125 | 50 |
| M | 1 | 67 | 26.3 | 41 | 101 | 100 | 60 | 110 | 56 |
| M | 2 | 67 | 26.9 | 48 | 104 | 100 |  |  | 65 |
| M | 1 | 71 | 34 | 42 | 133 | 119 | 80 | 150 | 55 |
| M | 2 | 52 | 33.8 | 43 | 117 | 113 | 80 | 130 | 60 |
| M | 1 | 60 | 24.2 | 48 | 110 | 100 | 80 | 120 | 60 |
| M | 2 | 59 | 24.8 | 39 | 97 | 105 |  |  | 0 |
| F | 1 | 68 | 43.9 | 44 | 129 | 126 | 80 | 115 | 0 |
| F | 2 | 68 | 40.6 | 44 | 141 | 127 | 100 | 180 | 0 |
| M | 1 | 72 | 35.6 | 44 | 123 | 109 | 80 | 140 | 70 |
| M | 2 | 72 | 36.8 |  |  |  |  |  | 0 |
| F | 1 | 57 | 54.7 |  |  |  |  |  | 0 |
| F | 2 | 45 | 48.4 | 49 | 135 | 124 |  |  | 35 |
| F | 1 | 67 | 48.4 |  |  |  |  |  | 0 |
| F | 2 | 66 | 47.8 | 38 | 144 | 139 | 100 | 150 | 0 |
| M | 1 | 67 | 27.1 | 45 | 100 | 110 | 80 | 135 | 0 |
| M | 2 | 69 | 28.7 | 39 | 110 | 111 | 80 | 130 | 0 |
| F | 1 | 56 | 52.7 |  |  |  |  |  | 0 |
| F | 2 | 57 | 50 |  |  |  |  |  | 30 |
| M | 1 | 78 | 25.4 | 39 | 101 | 103 | 70 | 120 | 0 |
| M | 2 | 82 | 26.5 | 41 | 104 | 99 | 70 | 100 | 35 |
| M | 1 | 58 | 37.6 | 49 | 139 | 119 | 90 | 120 | 50 |
| M | 2 | 57 | 36.2 | 45 | 126 | 125 | 80 | 115 | 40 |
| F | 1 | 74 | 34 | 39 | 114 | 120 | 90 | 140 | 45 |
| F | 2 | 74 | 32 | 39 | 109 | 109 | 75 | 115 | 0 |
| F | 1 | 70 | 34.4 | 34 | 126 | 114 | 80 | 125 | 17 |
| F | 2 | 70 | 33.2 | 34 | 124 | 119 | 80 | 120 | 0 |
| M | 1 | 63 | 31.1 | 48 | 125 | 93 | 80 | 120 | 60 |
| M | 2 | 62 | 31.6 | 45 | 113 | 118 | 80 | 120 | 30 |
| M | 1 | 58 | 33.3 | 40 | 118 | 100 | 80 | 120 | 20 |
| M | 2 | 57 | 32.1 | 43 | 113 | 107 | 90 | 120 | 0 |
| F | 1 | 76 | 41.6 | 39 | 129 | 92 | 70 | 130 | 0 |
| F | 2 | 73 | 37.5 | 39 | 133 | 124 | 80 | 130 | 0 |
| M | 3 | 37 | 36 | 39 | 120 | 111 | 60 | 105 | 20 |
| M | 3 | 30 | 27.4 | 41 | 91 | 103 | 80 | 120 |  |
| M | 3 | 37 | 29.3 | 38 | 113 | 116 | 70 | 105 | 15 |
| F | 3 | 53 | 24.6 | 36 | 88 | 93 | 80 | 120 | 0 |
| F | 3 | 48 | 28.7 | 36 | 102 | 109 | 100 | 150 | 0 |
| F | 3 | 45 | 37.9 | 38 | 115 | 125 | 80 | 140 | 30 |
| M | 3 | 39 | 26 | 40 | 99 | 107 | 80 | 120 | 5 |
| M | 3 | 34 | 34 | 41 | 104 | 104 | 90 | 130 | 20 |
| F | 3 | 56 | 33.3 | 34 | 110 | 108 | 80 | 120 | 30 |
| M | 3 | 59 | 37 | 44 | 117 | 102 | 95 | 120 | 0 |
| F | 3 | 56 | 24.6 | 34 | 105 | 113 | 65 | 120 | 0 |
| F | 3 | 61 | 25.3 | 33 | 84 | 109 | 95 | 135 | 0 |
| M | 3 | 44 | 30 | 48 | 102 | 111 | 120 | 140 | 20 |
| F | 3 | 57 | 29 | 34 | 99 | 117 | 80 | 110 | 40 |
| M | 3 | 51 | 28.1 | 39 | 110 | 95 | 90 | 140 | 25 |
| F | 3 | 78 | 38.7 | 37 | 115 | 128 | 80 | 140 | 25 |
| M | 3 | 56 | 43.8 | 44 | 136 | 137 |  |  | 20 |
| F | 3 | 61 | 34.9 | 40 | 108 | 111 | 80 | 120 | 25 |
| F | 3 | 76 | 31.2 | 34 | 105 | 115 | 85 | 115 | 0 |
| F | 3 | 57 | 33.3 | 36 | 111 | 113 | 80 | 125 | 25 |
| F | 3 | 63 | 25 | 35 | 80 | 100 | 80 | 110 | 0 |
| M | 3 | 56 | 29.4 | 44 | 105 | 106 | 80 | 130 | 0 |
| F | 3 | 45 | 37.9 | 38 | 115 | 125 | 80 | 140 | 30 |
| M | 3 | 63 | 29.4 | 43 | 109 | 107 | 80 | 130 | 60 |
| F | 3 | 52 | 35.1 | 33 | 102 | 119 | 90 | 140 | 0 |
| F | 3 | 73 | 32 | 35 | 112 | 108 | 80 | 120 | 0 |
| F | 3 | 45 | 32.7 | 35 | 108 | 113 |  |  | 25 |
| F | 3 | 51 | 35 | 37 | 98 | 129 |  |  | 0 |
| M | 3 | 51 | 43.3 | 43 | 135 | 128 |  |  | 35 |

| **FVC (lt)** | **FVC (%)** | **FEV1 (lt)** | **FEV1 (%)** | **FEV1/FVC** | **PEF (lt/minm)** | **PEF (%)** | **SaO2** |
| --- | --- | --- | --- | --- | --- | --- | --- |
| 3.15 | 77.44 | 2.23 | 67.93 | 70.68 | 5.88 | 70.72 | 92 |
| 4.31 | 109.1 | 3.8 | 121.8 | 88.28 | 8.27 | 102.4 | 97 |
| 2.7 | 55.16 | 1.7 | 44.1 | 63.01 | 4.36 | 47.78 | 91 |
| 4.03 | 98.7 | 3.42 | 105.3 | 84.95 | 8.53 | 103.3 | 97 |
| 3.29 | 73.41 | 1.87 | 52.4 | 56.82 | 4.28 | 49.14 | 98 |
| 5.28 | 96.96 | 4.49 | 99.03 | 85.1 | 8.72 | 85.99 | 95 |
| 2.86 | 69.07 | 2.14 | 66.92 | 74.61 | 6.7 | 82.21 | 96 |
| 3.12 | 86.6 | 2.47 | 88.87 | 79.12 | 8.19 | 108.3 | 95 |
| 2.76 | 83.63 | 1.87 | 65.93 | 67.87 | 3.67 | 54.94 | 97 |
| 2.99 | 92.11 | 2.47 | 88.45 | 82.53 | 5.95 | 89.98 | 93 |
| 2.06 | 77.56 | 1.67 | 74.31 | 81.06 | 6.06 | 102.6 | 97 |
|  |  |  |  |  |  |  | 91 |
| 2.04 | 80.29 | 1.56 | 73.21 | 76.11 | 3.41 | 58.72 | 93 |
| 2.64 | 102.39 | 2.17 | 100.88 | 82.18 | 3.23 | 55.33 |  |
|  |  |  | 38 |  |  |  |  |
|  |  |  |  |  |  |  |  |
| 3.73 | 79.12 | 2.65 | 73.11 | 70.9 |  |  |  |
| 4.03 | 109.5 | 3.4 | 119.5 | 84.35 | 4.54 | 59.29 | 98 |
| 2.14 | 63.46 | 1.18 | 47.08 | 55 | 1.42 | 19.91 | 95 |
| 1.52 | 47.62 |  |  |  | 3.26 | 46.53 | 95 |
| 1.86 | 76.86 | 1.32 | 65.75 | 70.65 | 3.92 | 69.29 | 92 |
| 2.57 | 108.7 | 2.2 | 112.9 | 85.68 | 5.51 | 98.82 | 98 |
| 3.61 | 86.2 | 2.49 | 78.13 | 69.15 | 6.44 | 79.08 | 97 |
| 3.19 | 77.84 | 2.65 | 84.65 | 83.27 | 5.86 | 72.56 | 96 |
| 3.04 | 78.34 | 2.29 | 73.25 | 75.41 | 5.57 | 68.93 | 97 |
| 4.09 | 96.38 | 3.65 | 106.9 | 89.09 | 10.87 | 128 | 97 |
| 3.87 | 80 | 2.76 | 72.27 | 71.14 | 9.15 | 101 | 96 |
| 3.6 | 78.16 | 3.29 | 90.38 | 91.44 | 5.05 | 57.29 | 96 |
| 2.87 | 60.93 | 2 | 55.07 | 69.72 | 7.58 | 86.17 | 97 |
| 4.25 | 90.48 | 3.45 | 93.29 | 81.23 | 9.4 | 105.7 | 95 |
| 2.94 | 83.04 | 2.14 | 78.19 | 72.64 | 6.81 | 90.81 | 94 |
|  |  |  |  |  |  |  |  |
| 2.6 | 72.32 | 2.02 | 74.91 | 77.94 | 5.83 | 78.37 | 95 |
| 2.71 | 82.05 | 2.19 | 88.51 | 80.59 | 4.25 | 59.79 | 97 |
| 2.56 | 64.04 | 2.29 | 71.56 | 89.37 | 5.48 | 67.02 | 91 |
| 3.85 | 87.97 | 3.17 | 91.46 | 82.45 | 7.47 | 87.16 | 97 |
| 1.61 | 54.92 | 1.03 | 46.46 | 64.03 | 2.69 | 39.81 | 94 |
| 2.91 | 80.59 | 2.13 | 77.18 | 73.33 | 5.72 | 75.86 |  |
| 1.86 | 81.54 | 1.43 | 74.89 | 76.58 | 4.81 | 88.28 | 95 |
|  |  |  |  |  |  |  |  |
| 3.74 | 84.65 | 2.67 | 77.26 | 71.35 | 6.41 | 75.05 | 96 |
| 2.98 | 74.78 | 2.88 | 91.97 | 96.91 | 8.13 | 100.5 | 97 |
| 1.63 | 51.46 | 1.06 | 43.5 | 65.2 | 5.36 | 75.62 | 88 |
| 3.24 | 89.32 | 2.69 | 96.56 | 83.05 | 7.8 | 102.97 | 96 |
| 2.62 | 70.7 | 1.8 | 62.67 | 68.74 | 5.55 | 71.97 | 97 |
| 2.27 | 66.39 | 1.73 | 64.88 | 76.1 | 5.22 | 70.5 | 95 |
| 3.01 | 71.98 | 1.84 | 57.72 | 61.2 | 4.34 | 53.2 | 96 |
| 2.43 | 64.96 | 2.06 | 71.73 | 84.76 | 6.39 | 83.03 | 96 |
| 1.37 | 30.5 | 1.27 | 36.52 | 93.05 | 2.27 | 26.46 | 98 |
| 4.12 | 99.42 | 3.06 | 93.26 | 74.2 | 9.57 | 115.3 | 98 |
| 2.11 | 71.35 | 1.67 | 66.86 | 78.89 | 5.01 | 79.41 | 95 |
| 2.52 | 103.5 | 2.2 | 109 | 87.48 | 6.25 | 110.6 | 95 |
| 1.84 | 46.27 | 1.23 | 40.62 | 66.85 | 3.02 | 38.06 | 93 |
|  |  |  |  |  |  |  |  |
| 2.41 | 78.62 | 1.64 | 62.81 | 67.94 | 3.66 | 57.02 | 97 |
| 2.57 | 74.01 | 2.21 | 73.88 | 85.99 | 4.76 | 69.04 | 96 |
| 1.8 | 73.62 | 1.24 | 60.68 | 68.64 |  |  | 93 |
| 2.26 | 91.02 | 1.81 | 87.29 | 79.99 | 4.56 | 79.89 | 95 |
| 2.88 | 67.2 | 1.98 | 59.81 | 68.68 | 4.2 | 50.53 | 97 |
| 3.72 | 100.2 | 3.2 | 111.7 | 85.87 | 5.43 | 70.67 | 96 |
| 2.37 | 82.26 | 1.73 | 70.9 | 73.11 | 3.37 | 54.59 | 97 |
| 2.08 | 77.97 | 1.67 | 74.13 | 80.27 | 6.84 | 115.4 | 95 |
| 2.72 | 74.4 | 2 | 73.19 | 73.48 | 5.9 | 78.93 | 95 |
| 2.47 | 89.98 | 1.86 | 92.5 | 75.37 | 2.3 | 35.59 | 98 |
| 2.05 | 53.54 | 1.08 | 35.29 | 52.54 | 3.42 | 42.87 | 92 |
| 3.38 | 72.58 | 2.7 | 73.91 | 79.81 | 6.61 | 74.48 | 96 |
| 2 | 71.11 | 1.48 | 63.05 | 74.09 | 3.81 | 62.17 | 95 |
| 1.83 | 74.49 | 1.6 | 79.04 | 87.79 | 2.81 | 49.4 | 96 |
| 2.39 | 100.5 | 1.65 | 83.91 | 69.13 | 2.93 | 52.5 | 95 |
| 2.31 | 98.9 | 1.84 | 95.05 | 79.51 | 3.92 | 70.77 | 98 |
| 1.66 | 42.22 | 0.94 | 30.63 | 56.84 | 2.62 | 32.79 | 92 |
| 3.21 | 83.08 | 2.85 | 93.48 | 88.79 | 7.89 | 99.07 | 98 |
| 4.12 | 91.06 | 2.89 | 80.94 | 70.17 | 7.66 | 88 | 96 |
| 2.91 | 73.22 | 2.45 | 77.52 | 84.45 | 7.44 | 91.42 | 96 |
| 1.28 | 63.31 | 1.12 | 67.86 | 87.14 | 3.15 | 60.99 | 92 |
| 2.07 | 93.61 | 1.59 | 87.72 | 76.96 | 4.47 | 82.99 | 98 |
| 3.39 | 80.68 | 3.2 | 90.49 | 94.28 | 5.45 | 62.77 | 96 |
| 5.36 | 108.1 | 4.23 | 101.6 | 78.91 | 11.85 | 123.4 | 98 |
| 4.67 | 91.75 | 3.67 | 87.18 | 78.49 | 8.53 | 88.39 | 95 |
| 3.32 | 108.3 | 2.80 | 106.9 | 84.22 | 6.87 | 107.3 | 96 |
| 3.22 | 104.6 | 2.81 | 106.3 | 87 | 6.97 | 108.7 | 98 |
| 3.55 | 120.7 | 2.86 | 113.5 | 80.47 | 5.69 | 91.28 | 98 |
| 5.7 | 104.2 | 4.82 | 108 | 84.52 | 12.79 | 127.7 | 98 |
| 5.07 | 106.9 | 4.36 | 110 | 86.03 | 9.83 | 105.6 | 98 |
| 2.38 | 103.6 | 2.06 | 107.1 | 86.52 | 6.22 | 113.9 | 98 |
| 3.98 | 103.1 | 3.15 | 102.9 | 79.27 | 9.41 | 117.8 | 96 |
| 3.01 | 94.54 | 2.8 | 103.1 | 92.99 | 6.74 | 102.8 | 97 |
| 3.27 | 115.5 | 2.4 | 100.4 | 73.36 | 4.66 | 75.93 | 97 |
| 5.62 | 122.3 | 4.42 | 117.5 | 78.6 | 8.74 | 97.07 | 95 |
| 3.38 | 114.9 | 2.93 | 117.6 | 86.87 | 7.8 | 124.7 | 98 |
| 5.3 | 118.5 | 4.41 | 122.6 | 83.26 | 12.95 | 147.8 | 98 |
| 2.34 | 107.65 | 1.82 | 102.94 | 78 | 4.63 | 86.62 | 94 |
| 3.58 | 73.64 | 3.07 | 79.85 | 85.69 | 7.28 | 79.99 | 98 |
| 2.79 | 111.2 | 2.25 | 107 | 80.73 | 5.43 | 94.83 | 92 |
| 1.88 | 84.43 | 1.51 | 82.91 | 80.44 | 3.91 | 72.22 | 96 |
| 2.92 | 119 | 2.23 | 108.4 | 76.51 | 5.49 | 97.15 | 95 |
| 3.29 | 140.6 | 2.79 | 143.1 | 84.71 | 6.73 | 121.8 | 96 |
| 4.59 | 103.1 | 3.88 | 109.7 | 84.56 | 9.04 | 104.3 | 97 |
| 3.55 | 120.7 | 2.86 | 113.5 | 80.47 | 5.69 | 91.28 | 98 |
| 3.18 | 78.1 | 2.63 | 82.19 | 82.57 | 7.81 | 95.62 | 96 |
| 2.99 | 124.64 | 2.44 | 120.4 | 81.4 | 5.72 | 102.51 | 99 |
| 1.32 | 57.17 | 1.12 | 58.94 | 84.94 | 2.61 | 47.4 | 97 |
| 2.9 | 88.04 | 2.53 | 89.3 | 87.21 | 4.44 | 66.52 | 98 |
| 3.52 | 117 | 3.04 | 118.7 | 86.49 | 6.65 | 105.2 | 98 |
| 3.72 | 79.05 | 3.32 | 87.93 | 89.21 | 9.31 | 103.3 | 99 |

| **ESS** | **FSS** | **HADS A** | **HADS D** | **TRT (min)** | **TST (min)** | **SO (min)** | **SE (%)** |
| --- | --- | --- | --- | --- | --- | --- | --- |
| 4 | 1.5 | 5 | 4 | 243.5 | 166 | 67.5 | 68.2 |
| 12 | 4.1 | 3 | 3 | 174.4 | 116.5 | 27.8 | 66.7 |
| 4 | 5.4 | 13 | 10 | 342.4 | 273 | 21.8 | 79.7 |
| 2 | 4 | 2 | 1 | 419.9 | 287.2 | 23.7 | 68.4 |
| 9 | 3.5 | 1 | 2 | 389.7 | 273.1 | 20.1 | 70.1 |
| 7 | 4 | 1 | 1 | 180.1 | 53.7 | 122.3 | 29.8 |
| 12 | 5.1 | 1 | 7 | 346 | 189.5 | 10 | 55.7 |
|  |  |  |  | 383.8 | 309.8 | 18 | 80.7 |
| 9 | 6.3 | 5 | 8 | 182.7 | 141.9 | 22.6 | 77.7 |
| 8 | 3.4 | 2 | 4 | 415.6 | 397.6 | 5 | 95.7 |
| 14 |  |  |  | 206 | 182.5 | 17 | 88.6 |
|  |  |  |  | 433.5 | 364.6 | 18.9 | 84.1 |
| 21 | 3 | 4 | 0 | 187.5 | 165.6 | 5.5 | 88.3 |
|  |  |  |  | 440.7 | 369.5 | 3.5 | 83.8 |
|  |  |  |  | 435.3 | 241.5 | 13 | 57.2 |
|  |  |  |  | 182.8 | 179.8 | 1.5 | 98.4 |
|  |  |  |  | 220.2 | 185 | 11.7 | 84 |
| 8 |  |  |  | 373.5 | 363.4 | 5.7 | 97.3 |
|  |  |  |  | 391 | 339.5 | 4 | 87.5 |
|  |  |  |  | 364 | 284.5 | 3 | 78.1 |
|  |  |  |  | 431.4 | 200 | 8 | 46.4 |
| 11 | 3 | 5 | 7 | 423 | 267.5 | 86 | 63.2 |
| 6 | 6 | 10 | 6 | 228.3 | 199.4 | 10 | 99.5 |
|  |  |  |  | 329.2 | 242.7 | 3.5 | 73.7 |
|  |  |  |  | 370.2 | 327.5 | 1 | 88.5 |
| 18 | 4.3 | 8 | 8 | 179 | 171 | 2 | 98.3 |
| 7 | 2.3 | 5 | 5 | 163.7 | 151.2 | 4.5 | 92.4 |
| 11 | 4.3 | 9 | 7 | 408.4 | 360.6 | 47.8 | 88.3 |
| 4 | 2 | 1 | 2 | 412 | 281 | 16 | 68.7 |
| 11 | 5.4 | 4 | 5 | 396.8 | 269.8 | 7.5 | 68 |
| 16 | 3 | 1 | 7 | 183.4 | 177 | 6.3 | 96.5 |
|  |  |  |  | 209.7 | 154.4 | 28.3 | 73.6 |
|  |  |  |  | 381 | 372.3 | 8.7 | 96.3 |
|  |  |  |  | 416.2 | 281.3 | 79.4 | 67.6 |
| 15 | 7 | 7 | 7 | 199 | 191 | 6 | 97 |
| 7 | 5 | 0 | 3 | 168.7 | 95.6 | 15.1 | 56.7 |
| 15 | 4.7 | 0 | 0 |  | 299 |  |  |
|  |  |  |  | 231.2 | 133.7 | 45 | 57.8 |
| 6 | 2.5 | 12 | 5 | 409.4 | 391 | 5 | 95.5 |
|  |  |  |  | 257 | 253.5 | 2.5 | 98.6 |
| 9 | 2.4 | 7 | 9 | 361.7 | 346.8 | 0.5 | 95.9 |
| 7 | 2 | 3 | 4 | 441.9 | 275.9 | 12.5 | 62.4 |
| 14 | 5.6 | 10 | 9 | 442.8 | 266.8 | 34 | 60.3 |
| 22 | 4 | 5 | 2 | 119.8 | 117.5 | 0.7 | 98.2 |
| 12 |  |  |  | 418.5 | 288.5 | 5 | 68.9 |
| 15 | 5.8 |  |  | 237 | 120 | 19.5 | 50.6 |
| 12 | 1.1 | 1 | 3 | 119.7 | 106.2 | 13.5 | 88.7 |
| 2 | 3.6 | 8 | 4 | 391.7 | 297.5 | 39 | 76 |
|  |  |  |  | 393.3 | 348 | 13.5 | 88.5 |
| 15 | 2 |  |  | 400.1 | 391.5 | 3.5 | 97.8 |
| 12 | 6.1 | 0 | 5 | 417.1 | 385.6 | 31.5 | 33.7 |
| 9 | 5 | 4 | 8 | 167 | 134 | 18.5 | 80 |
| 24 | 7 | 10 | 7 | 213 | 114 | 30 | 53.5 |
|  |  |  |  | 288.3 | 228.8 | 7.5 | 79.4 |
| 12 |  |  |  | 448.2 | 331.7 | 29.5 | 74 |
| 9 | 5.6 | 10 | 8 | 383.4 | 346 | 4.5 | 90.2 |
|  |  |  |  | 222.6 | 88 | 22.2 | 39.5 |
| 4 | 7 | 4 | 2 | 167.1 | 143.3 | 14.9 | 85.7 |
|  |  |  |  | 374.2 | 312 | 1.2 | 83.4 |
| 16 | 5.4 | 0 | 0 | 150 | 101.8 | 9.7 | 67.9 |
| 14 | 3.4 |  |  | 189.2 | 172.3 | 17 | 91 |
|  |  |  |  | 214 | 164.4 | 22 | 76.7 |
| 7 | 5.2 | 0 | 2 | 427 | 351 | 6.5 | 84.3 |
| 17 | 5.7 |  |  | 412.4 | 48 | 4.5 | 11.6 |
| 9 | 5 | 7 | 7 | 162.7 | 151.8 | 10.9 | 93.3 |
| 9 | 3 | 2 | 4 | 123 | 111 | 8.7 | 90.2 |
| 11 | 4 | 0 | 2 | 182.6 | 141.8 | 21.8 | 77.6 |
| 13 |  |  |  | 390.1 | 313.6 | 26 | 80.4 |
| 7 | 6.7 | 8 | 8 | 442.7 | 264.8 | 42.9 | 59.8 |
| 4 | 3.7 | 6 | 5 | 454.2 | 302.2 | 38.5 | 66.5 |
| 11 | 6.2 | 1 | 11 | 354.7 | 354.7 | 0 | 100 |
| 6 | 1 | 1 | 2 | 197.6 | 181.6 | 5.5 | 81.7 |
| 6 | 3 | 5 | 9 | 352.5 | 339.5 | 13 | 73 |
| 9 | 8 | 6 | 9 | 128.4 | 120.8 | 3.5 | 94.1 |
| 16 | 6.6 | 5 | 2 | 140.9 | 73.4 | 9.5 | 52.1 |
| 10 | 5.7 | 4 | 6 | 413 | 375.5 | 12 | 90.9 |
| 8 | 4.3 | 6 | 3 | 395.2 | 330 | 55.5 | 83.5 |
| 4 | 1 | 1 | 0 | 377.4 | 355.4 | 11 | 94.2 |
| 4 | 1.2 | 1 | 3 |  |  |  |  |
| 8 | 6.3 | 8 | 2 | 344.5 | 129 | 42.5 | 37.4 |
| 12 | 5.1 | 6 | 6 | 346 | 334 | 6.5 | 96.5 |
| 5 | 5.7 | 18 | 17 | 392.8 | 346.3 | 46.5 | 77.7 |
|  |  |  |  | 361.8 | 319.5 | 34.5 | 88.3 |
| 10 | 5.7 | 4 | 6 | 404.5 | 304 | 28 | 75.1 |
| 13 | 6.8 | 13 | 12 | 521 | 226.5 | 96.5 | 54.6 |
| 0 | 3.6 | 8 | 7 | 357.5 | 343 | 14.5 | 95.9 |
| 19 | 4.8 | 8 | 10 | 404.7 | 254 | 13.2 | 62.8 |
| 0 |  | 12 | 8 | 400.2 | 376.7 | 11.5 | 94.1 |
| 9 | 5.5 | 6 | 8 | 420.3 | 419.3 | 0.7 | 99.6 |
| 5 | 3.4 | 7 | 4 | 388.5 | 372.8 | 8.2 | 96 |
| 7 | 4.3 | 11 | 8 | 377.4 | 289 | 62.8 | 76.6 |
| 2 | 5.6 | 13 | 5 | 415.1 | 366.2 | 7.4 | 88.2 |
| 6 | 2 | 9 | 4 | 248 | 193 | 2.5 | 77.8 |
| 10 | 3 | 7 | 7 | 441.5 | 414.9 | 18.1 | 94 |
| 7 | 4.2 | 1 | 3 | 370.2 | 192.5 | 148 | 52 |
| 9 | 5.6 | 8 | 9 | 369.5 | 356 | 7 | 96.4 |
| 4 | 4 | 7 | 7 | 387.6 | 295.5 | 27.8 | 76.2 |
| 1 | 2.1 | 3 | 1 | 393.1 | 191 | 72.5 | 48.6 |
| 5 | 5.7 | 18 | 17 | 392.8 | 346.3 | 46.5 | 77.7 |
| 20 | 2.3 | 12 | 7 | 397.4 | 308.6 | 58 | 77.6 |
| 7 | 5.4 | 1 | 12 | 385.1 | 259.5 | 45.5 | 67.4 |
| 7 | 5.7 | 10 | 11 | 418.3 | 286.5 | 35.5 | 68.5 |
| 8 | 4 | 10 | 6 | 428 | 395 | 10 | 92.9 |
| 9 | 5.6 | 2 | 9 | 380 | 357 | 13.1 | 93.9 |
| 7 | 4.2 |  |  | 388.7 | 380.7 | 4 | 97.9 |

| **S1 (min)** | | **S1 (%)** | **S2 (min)** | | | **S2 (%)** | **S3-4 (min)** | | **S3-4 (%)** | | **REM (min)** | **REM (%)** | |
| --- | --- | --- | --- | --- | --- | --- | --- | --- | --- | --- | --- | --- | --- |
| 10 | 5.7 | | 98 | | | 59 | 22 | | 13.5 | | 36 | 21.7 | |
| 6.5 | 5.6 | | 110 | | | 94.4 | 0 | | 0 | | 0 | 0 | |
| 7 | 2.6 | | 266 | | | 97.4 | 0 | | 0 | | 0 | 0 | |
| 10 | 3.5 | | 211.5 | | | 73.7 | 0 | | 0 | | 65.7 | 22.9 | |
| 19.5 | 7.1 | | 242.5 | | | 88.8 | 0 | | 0 | | 11.1 | 4.1 | |
| 6.5 | 12.1 | | 47.2 | | | 87.9 | 0 | | 0 | | 0 | 0 | |
| 18 | 9.5 | | 171.5 | | | 90.5 | 0 | | 0 | | 0 | 0 | |
| 30 | 9.7 | | 204.5 | | | 66 | 13.5 | | 4.4 | | 61.8 | 19.9 | |
| 4 | 2.8 | | 137.9 | | | 97.2 | 0 | | 0 | | 0 | 0 | |
| 138.5 | 34.8 | | 150.5 | | | 37.9 | 76.5 | | 19.2 | | 32.1 | 8.1 | |
| 35.5 | 19.5 | | 69.5 | | | 38.1 | 77.5 | | 42.5 | | 0 | 0 | |
| 13 | 3.6 | | 321.1 | | | 88.1 | 0 | | 0 | | 30.5 | 8.4 | |
| 42.1 | 25.4 | | 94.5 | | | 57.1 | 11.5 | | 6.9 | | 17.5 | 10.6 | |
| 24.5 | 6.6 | | 140.5 | | | 38 | 130.5 | | 35.3 | | 74 | 20 | |
| 82.5 | 34.2 | | 132 | | | 54.7 | 0 | | 0 | | 27 | 11.2 | |
| 0.5 | 0.3 | | 9.5 | | | 5.3 | 163.3 | | 90.8 | | 6.5 | 3.6 | |
| 28.5 | 15.4 | | 95 | | | 51.4 | 48 | | 25.9 | | 13.5 | 7.3 | |
| 12.5 | 3.4 | | 302.9 | | | 83.3 | 0 | | 0 | | 48 | 13.2 | |
| 18 | 5.3 | | 204.5 | | | 60.2 | 79 | | 23.3 | | 38 | 11.2 | |
| 46.5 | 16.3 | | 104.5 | | | 36.7 | 80 | | 28.1 | | 53.5 | 18.8 | |
| 119 | 59.5 | | 67.5 | | | 33.8 | 13.5 | | 6.7 | | 0 | 0 | |
| 52 | 19.4 | | 84.5 | | | 31.6 | 43 | | 17.3 | | 72.5 | 27.1 | |
| 87.9 | 44.1 | | 37 | | | 18.6 | 74.5 | | 37.4 | | 0 | 0 | |
| 88 | 36.3 | | 76.2 | | | 31.4 | 50 | | 20.6 | | 28.5 | 11.7 | |
| 74 | 22.6 | | 206.5 | | | 63.1 | 9 | | 2.7 | | 38 | 11.6 | |
| 2 | 1.2 | | 160 | | | 93.6 | 0 | | 0 | | 9 | 5.3 | |
| 46.5 | 30.8 | | 33.5 | | | 22.2 | 45.6 | | 30.2 | | 25.5 | 16.9 | |
| 1.5 | 0.4 | | 348.6 | | | 96.7 | 0 | | 0 | | 10.5 | 2.9 | |
| 14.5 | 5.2 | | 257 | | | 91.5 | 0 | | 0 | | 9.5 | 3.4 | |
| 68.5 | 25.8 | | 172.5 | | | 63.9 | 19.5 | | 7.2 | | 8.3 | 3.1 | |
| 2 | 1.1 | | 148.5 | | | 83.9 | 0 | | 0 | | 26.5 | 15 | |
| 102.4 | 67 | | 52 | | | 33.7 | 0 | | 0 | | 0 | 0 | |
| 7 | 1.9 | | 325.3 | | | 88.7 | 0 | | 0 | | 34.5 | 9.4 | |
| 23 | 8.2 | | 234.3 | | | 83.3 | 0 | | 0 | | 24 | 8.5 | |
| 2.5 | 1.3 | | 139 | | | 72.8 | 49.5 | | 25.9 | | 0 | 0 | |
| 32.1 | 33.6 | | 54 | | | 56.5 | 9.5 | | 9.9 | | 0 | 0 | |
| 166.5 | 55.5 | | 84 | | | 79 | 48.5 | | 19.5 | | 0 | 0 | |
| 0.5 | 0.4 | | 124.7 | | | 93.3 | 8.5 | | 6.8 | | 0 | 0 | |
| 64 | 16.4 | | 155 | | | 39.8 | 108.5 | | 27.7 | | 63 | 16.1 | |
| 6 | 2.4 | | 125 | | | 49.3 | 91.5 | | 36.1 | | 31 | 12.2 | |
| 79.5 | 22.9 | | 160.5 | | | 46.3 | 56 | | 16.1 | | 50.8 | 14.6 | |
| 0 | 0 | | 35 | | | 12.7 | 229.8 | | 83.3 | | 11 | 4 | |
| 0 | 0 | | 175.8 | | | 65.9 | 34.5 | | 12.9 | | 56.5 | 21.2 | |
| 2.5 | 2.1 | | 103.5 | | | 88.1 | 11.5 | | 9.8 | | 0 | 0 | |
| 39.5 | 13.7 | | 160.5 | | | 55.6 | 60.5 | | 21 | | 28 | 9.7 | |
| 13.5 | 11.3 | | 10.5 | | | 8.8 | 96 | | 80 | | 0 | 0 | |
| 2 | 1.9 | | 96.2 | | | 90.6 | 8 | | 7.5 | | 0 | 0 | |
| 0 | 0 | | 119 | | | 40 | 105 | | 35.3 | | 73.5 | 24.7 | |
| 63 | 18.1 | | 143 | | | 41.1 | 132 | | 37 | | 10 | 2.9 | |
| 39.5 | 10.1 | | 163.5 | | | 41.8 | 86.5 | | 22.1 | | 102 | 26.1 | |
| 16.5 | 11.7 | | 64 | | | 45.5 | 38 | | 27 | | 22.1 | 15.7 | |
| 5 | 3.7 | | 49.5 | | | 37.1 | 79 | | 59.2 | | 0 | 0 | |
| 18 | 15.8 | | 68.5 | | | 60.1 | 27.5 | | 24.1 | | 0 | 0 | |
| 63 | 27.5 | | 124.8 | | | 54.5 | 0 | | 0 | | 41 | 17.9 | |
| 79 | 23.8 | | 156.7 | | | 47.2 | 40 | | 12.1 | | 56 | 16.9 | |
| 121 | 36 | | 139 | | | 40.2 | 61 | | 17.6 | | 25 | 7.2 | |
| 40 | 45.5 | | 45 | | | 51.1 | 3 | | 3.4 | | 0 | 0 | |
| 7.5 | 5.2 | | 135.8 | | | 94.8 | 0 | | 0 | | 0 | 0 | |
| 7 | 2.2 | | 290.5 | | | 93.1 | 0 | | 0 | | 14.5 | 4.6 | |
| 21.5 | 21.1 | | 80.3 | | | 78.9 | 0 | | 0 | | 0 | 0 | |
| 26 | 15.1 | | 74.3 | | | 43.1 | 72 | | 41.8 | | 0 | 0 | |
| 16 | 9.7 | | 98.5 | | | 59.9 | 49.9 | | 30.3 | | 0 | 0 | |
| 6.5 | 1.9 | | 288 | | | 82.1 | 0 | | 0 | | 56.5 | 16.1 | |
| 48 | 100 | | 0 | | | 0 | 0 | | 0 | | 0 | 0 | |
| 3.5 | 2.3 | | 148.3 | | | 97.7 | 0 | | 0 | | 0 | 0 | |
| 6 | 5.4 | | 105 | | | 94.6 | 0 | | 0 | | 0 | 0 | |
| 7.5 | 5.3 | | 95.8 | | | 67.5 | 38.5 | | 27.1 | | 0 | 0 | |
| 62 | 19.8 | | 156.1 | | | 49.8 | 59 | | 18.8 | | 36.5 | 11.6 | |
| 17 | 6.4 | | 221.3 | | | 83.6 | 0 | | 0 | | 26.5 | 10 | |
| 36 | 11.9 | | 94.7 | | | 31.3 | 63 | | 20.8 | | 108.5 | 35.9 | |
| 0 | 0 | | 236 | | | 66.5 | 69.5 | | 19.6 | | 49.2 | 13.9 | |
| 0 | 0 | | 67 | | | 36.9 | 101.5 | | 55.9 | | 13 | 7.2 | |
| 0 | 0 | | 74.5 | | | 28.9 | 131 | | 50.9 | | 52 | 20.2 | |
| 5 | 4.1 | | 115.8 | | | 95.9 | 0 | | 0 | | 0 | 0 | |
| 11 | 15 | | 62.4 | | | 85 | 0 | | 0 | | 0 | 0 | |
| 0 | 0 | | 162.5 | | | 43.3 | 137 | | 36.5 | | 76 | 20.2 | |
| 3 | 0.9 | | 154 | | | 46.7 | 143 | | 43.3 | | 30 | 9.1 | |
| 7.5 | 2.1 | | 239.4 | | | 67.4 | 77.5 | | 21.8 | | 31 | 8.7 | |
|  |  | |  | | |  |  | |  | |  |  | |
| 2 | 1.6 | | 72 | | | 55.8 | 55 | | 42.6 | | 0 | 0 | |
| 6.5 | 1.9 | | 137.5 | | | 41.2 | 154.5 | | 46.3 | | 35.5 | 10.5 | |
| 9 | 3 | | 115 | | | 37.7 | 119 | | 39 | | 62 | 20.3 | |
| 0 | 0 | | 125.5 | | | 39.3 | 120 | | 37.6 | | 74 | 23.2 | |
| 29.5 | 9.7 | | 139 | | | 45.7 | 113 | | 37.2 | | 22.5 | 7.4 | |
| 12.5 | 5.5 | | 149.5 | | | 66 | 45 | | 19.9 | | 19.5 | 8.6 | |
| 2 | 0.6 | | 232.5 | | | 67.8 | 70.5 | | 20.6 | | 38 | 11.1 | |
| 18.5 | 7.3 | | 209.2 | | | 82.5 | 26 | | 10.2 | | 0 | 0 | |
| 6.5 | 31.3 | | 370.2 | | | 98.3 | 0 | | 0 | | 0 | 0 | |
| 52 | 12.4 | | 230.8 | | | 55 | 65.5 | | 15.6 | | 71 | 16.9 | |
| 4.5 | 1.2 | | 212.3 | | | 57 | 65.5 | | 17.6 | | 90.5 | 24.3 | |
| 20 | 6.9 | | 187.5 | | | 64.9 | 39.5 | | 13.7 | | 42 | 14.5 | |
| 10 | 2.7 | | 33.7.7 | | | 92.2 | 0 | | 0 | | 18.5 | 5.1 | |
| 41 | 21.5 | | 132.5 | | | 68.7 | 10 | | 5.2 | | 9.5 | 4.9 | |
| 10.5 | 2.5 | | 368.4 | | | 88.8 | 36 | | 8.7 | | 0 | 0 | |
| 8 | 4.2 | | 28 | | | 14.5 | 122 | | 63.4 | | 34.5 | 17.9 | |
| 6 | 1.7 | | 314.5 | | | 88.3 | 0 | | 0 | | 35.5 | 10 | |
| 16.5 | 5.6 | | 194.5 | | | 65.8 | 64.5 | | 21.8 | | 20 | 6.8 | |
| 6.5 | 3.4 | | 75.5 | | | 39.5 | 91 | | 47.6 | | 18 | 9.4 | |
| 9 | 3 | | 115 | | | 37.7 | 119 | | 39 | | 62 | 20.3 | |
| 17.3 | 5.7 | | 279.1 | | | 90.4 | 0 | | 0 | | 12 | 3.9 | |
| 0 | 0 | | 26 | | | 10 | 198.5 | | 76.5 | | 35 | 13.5 | |
| 9.5 | 3.3 | | 84.5 | | | 29.5 | 174 | | 60.7 | | 18.5 | 6.5 | |
| 5.5 | 1.4 | | 304 | | | 77 | 60.5 | | 17.6 | | 90.5 | 15.3 | |
| 8 | 2.2 | | 215 | | | 60.2 | 73.5 | | 20.6 | | 60.5 | 16.9 | |
| 32 | 8.4 | | 109.7 | | | 28.8 | 181.5 | | 47.7 | | 57.7 | 15.1 | |
| **APNEAS/HYPOPNEAS (N)** | | | | | **OBSTRUCTIVE** | | **CENTRAL** | **MIXED** | | **HYPOPNEAS** | | |  |
| 321 | | | | | 190 | | 8 | 5 | | 118 | | |  |
| 158 | | | | | 116 | | 0 | 0 | | 33 | | |  |
| 443 | | | | | 253 | | 2 | 6 | | 180 | | |  |
| 182 | | | | | 147 | | 1 | 1 | | 33 | | |  |
| 121 | | | | | 0 | | 18 | 0 | | 103 | | |  |
| 56 | | | | | 6 | | 41 | 0 | | 9 | | |  |
| 205 | | | | | 140 | | 14 | 32 | | 19 | | |  |
| 102 | | | | | 0 | | 23 | 0 | | 79 | | |  |
| 56 | | | | | 1 | | 3 | 0 | | 52 | | |  |
| 99 | | | | | 19 | | 2 | 9 | | 69 | | |  |
| 108 | | | | | 1 | | 2 | 0 | | 105 | | |  |
| 74 | | | | | 2 | | 10 | 0 | | 62 | | |  |
| 262 | | | | | 259 | | 0 | 1 | | 2 | | |  |
| 374 | | | | | 211 | | 1 | 4 | | 158 | | |  |
| 137 | | | | | 41 | | 12 | 38 | | 46 | | |  |
| 144 | | | | | 51 | | 0 | 0 | | 91 | | |  |
| 41 | | | | | 12 | | 2 | 23 | | 4 | | |  |
| 249 | | | | | 60 | | 24 | 17 | | 148 | | |  |
| 182 | | | | | 100 | | 45 | 5 | | 150 | | |  |
| 85 | | | | | 4 | | 1 | 2 | | 78 | | |  |
| 230 | | | | | 23 | | 1 | 3 | | 203 | | |  |
| 284 | | | | | 220 | | 0 | 0 | | 63 | | |  |
| 98 | | | | | 18 | | 14 | 4 | | 62 | | |  |
| 127 | | | | | 41 | | 0 | 0 | | 86 | | |  |
| 321 | | | | | 165 | | 2 | 32 | | 62 | | |  |
| 120 | | | | | 70 | | 0 | 1 | | 49 | | |  |
| 37 | | | | | 7 | | 0 | 0 | | 30 | | |  |
| 77 | | | | | 1 | | 24 | 0 | | 52 | | |  |
| 326 | | | | | 37 | | 114 | 16 | | 159 | | |  |
| 262 | | | | | 229 | | 10 | 5 | | 18 | | |  |
| 39 | | | | | 3 | | 1 | 0 | | 35 | | |  |
| 137 | | | | | 118 | | 1 | 18 | | 79 | | |  |
| 195 | | | | | 7 | | 56 | 1 | | 0 | | |  |
| 244 | | | | | 5 | | 121 | 57 | | 121 | | |  |
| 209 | | | | | 127 | | 14 | 2 | | 66 | | |  |
| 160 | | | | | 93 | | 34 | 7 | | 26 | | |  |
| 208 | | | | | 10 | | 2 | 1 | | 195 | | |  |
| 254 | | | | | 62 | | 0 | 0 | | 192 | | |  |
| 292 | | | | | 67 | | 0 | 0 | | 225 | | |  |
| 91 | | | | | 36 | | 0 | 6 | | 51 | | |  |
| 254 | | | | | 78 | | 1 | 0 | | 175 | | |  |
| 124 | | | | | 86 | | 27 | 5 | | 6 | | |  |
| 93 | | | | | 74 | | 0 | 0 | | 19 | | |  |
| 147 | | | | | 147 | | 0 | 0 | | 0 | | |  |
| 235 | | | | | 164 | | 0 | 0 | | 71 | | |  |
| 89 | | | | | 65 | | 0 | 4 | | 20 | | |  |
| 109 | | | | | 6 | | 74 | 2 | | 87 | | |  |
| 105 | | | | | 27 | | 3 | 1 | | 74 | | |  |
| 366 | | | | | 165 | | 68 | 49 | | 84 | | |  |
| 141 | | | | | 84 | | 2 | 0 | | 55 | | |  |
| 69 | | | | | 25 | | 0 | 0 | | 44 | | |  |
| 108 | | | | | 6 | | 9 | 0 | | 99 | | |  |
| 110 | | | | | 94 | | 0 | 0 | | 16 | | |  |
| 263 | | | | | 211 | | 11 | 8 | | 33 | | |  |
| 24 | | | | | 0 | | 0 | 0 | | 24 | | |  |
| 70 | | | | | 25 | | 0 | 0 | | 45 | | |  |
| 84 | | | | | 82 | | 0 | 0 | | 2 | | |  |
| 85 | | | | | 5 | | 8 | 3 | | 69 | | |  |
| 106 | | | | | 34 | | 0 | 0 | | 72 | | |  |
| 100 | | | | | 30 | | 49 | 1 | | 20 | | |  |
| 329 | | | | | 124 | | 0 | 0 | | 205 | | |  |
| 246 | | | | | 65 | | 0 | 0 | | 181 | | |  |
| 163 | | | | | 121 | | 8 | 0 | | 34 | | |  |
| 67 | | | | | 3 | | 1 | 0 | | 63 | | |  |
| 286 | | | | | 70 | | 36 | 0 | | 180 | | |  |
| 137 | | | | | 79 | | 19 | 0 | | 39 | | |  |
| 66 | | | | | 36 | | 9 | 0 | | 21 | | |  |
| 163 | | | | | 59 | | 0 | 0 | | 104 | | |  |
| 107 | | | | | 46 | | 0 | 0 | | 61 | | |  |
| 156 | | | | | 73 | | 3 | 0 | | 80 | | |  |
| 574 | | | | | 79 | | 18 | 0 | | 477 | | |  |
| 203 | | | | | 189 | | 1 | 0 | | 13 | | |  |
| 74 | | | | | 16 | | 0 | 0 | | 58 | | |  |
| 132 | | | | | 27 | | 4 | 3 | | 98 | | |  |
| 123 | | | | | 2 | | 63 | 1 | | 67 | | |  |
| 463 | | | | | 321 | | 36 | 20 | | 86 | | |  |
| 16 | | | | | 0 | | 3 | 0 | | 13 | | |  |
| 37 | | | | | 0 | | 5 | 0 | | 32 | | |  |
|  | | | | |  | |  |  | |  | | |  |
| 9 | | | | | 3 | | 0 | 0 | | 6 | | |  |
| 33 | | | | | 0 | | 0 | 0 | | 33 | | |  |
| 20 | | | | | 6 | | 0 | 0 | | 14 | | |  |
| 17 | | | | | 5 | | 1 | 0 | | 11 | | |  |
| 45 | | | | |  | |  |  | |  | | |  |
| 26 | | | | | 22 | | 2 | 0 | | 2 | | |  |
| 60 | | | | | 16 | | 21 | 0 | | 44 | | |  |
| 3 | | | | | 0 | | 0 | 0 | | 3 | | |  |
| 54 | | | | | 28 | | 0 | 0 | | 26 | | |  |
| 82 | | | | | 13 | | 6 | 0 | | 63 | | |  |
| 35 | | | | | 8 | | 7 | 0 | | 20 | | |  |
| 23 | | | | | 1 | | 1 | 0 | | 22 | | |  |
| 68 | | | | |  | |  |  | |  | | |  |
| 32 | | | | | 30 | | 0 | 1 | | 1 | | |  |
| 20 | | | | |  | |  |  | |  | | |  |
| 16 | | | | | 0 | | 0 | 0 | | 16 | | |  |
| 37 | | | | |  | |  |  | |  | | |  |
| 15 | | | | | 1 | | 10 | 0 | | 4 | | |  |
| 3 | | | | | 0 | | 2 | 0 | | 1 | | |  |
| 20 | | | | | 6 | | 0 | 0 | | 14 | | |  |
| 49 | | | | | 1 | | 6 | 1 | | 41 | | |  |
| 15 | | | | | 0 | | 0 | 0 | | 15 | | |  |
| 12 | | | | | 2 | | 1 | 0 | | 9 | | |  |
| 5 | | | | | 0 | | 2 | 0 | | 3 | | |  |
| 37 | | | | | 0 | | 12 | 0 | | 25 | | |  |
| 80 | | | | | 4 | | 0 | 0 | | 76 | | |  |
| **MEAN DURATION (sec)** | | | | **MAX DURATION (sec)** | | | **AHI** | **AHI (nREM)** | | **AHI (REM)** | | |  |
| 17.3 | | | | 59.6 | | | 116 | 122.3 | | 93.3 | | |  |
| 18.5 | | | | 38.7 | | | 81.4 | 81.4 | |  | | |  |
| 16.5 | | | | 39 | | | 97.4 | 97.4 | | 0 | | |  |
| 30.2 | | | | 70.1 | | | 38.2 | 36.6 | | 43 | | |  |
| 18.7 | | | | 40.1 | | | 26 | 26.3 | | 32.4 | | |  |
| 15.9 | | | | 27.4 | | | 62.5 | 62.5 | |  | | |  |
| 21.1 | | | | 47 | | | 64.9 | 64.9 | |  | | |  |
| 22.7 | | | | 36.4 | | | 19.8 | 21.3 | | 13.6 | | |  |
| 23.5 | | | | 47 | | | 33.7 | 33.7 | |  | | |  |
| 15.6 | | | | 60.6 | | | 14.9 | 14.6 | | 18.7 | | |  |
| 12.8 | | | | 22.4 | | | 35.5 | 35.5 | |  | | |  |
| 19.8 | | | | 35 | | | 12.2 | 10.8 | | 27.5 | | |  |
| 28 | | | | 58.2 | | | 94.9 | 96.8 | | 78.9 | | |  |
| 18.3 | | | | 81.7 | | | 60.7 |  | |  | | |  |
| 23.6 | | | | 48.7 | | | 34.7 |  | |  | | |  |
| 33.8 | | | | 133.4 | | | 48.1 | 47.8 | | 55.4 | | |  |
| 25.3 | | | | 67.8 | | | 13.3 |  | |  | | |  |
| 23.2 | | | | 59.9 | | | 41.1 | 39.2 | | 53.8 | | |  |
| 26.6 | | | | 62 | | | 32.2 | 31.6 | | 36.3 | | |  |
| 39.7 | | | | 64.7 | | | 17.9 | 18.2 | | 16.8 | | |  |
| 19.9 | | | | 45.4 | | | 69 | 69 | |  | | |  |
| 21.3 | | | | 41.5 | | | 63.7 | 61.4 | | 71.8 | | |  |
| 19.3 | | | | 38.3 | | | 29.5 | 29.5 | |  | | |  |
| 28.8 | | | | 52.1 | | | 31.4 | 32.5 | | 23.2 | | |  |
| 25.8 | | | | 50.1 | | | 58.8 | 56.2 | | 78.9 | | |  |
| 24.9 | | | | 50.5 | | | 42.1 | 40.4 | | 73.3 | | |  |
| 17.4 | | | | 31.6 | | | 14.7 |  | |  | | |  |
| 22.8 | | | | 51.4 | | | 12.8 | 12.3 | | 28.6 | | |  |
| 21.1 | | | | 38 | | | 69.6 | 70.1 | | 56.8 | | |  |
| 14.2 | | | | 60 | | | 58.3 |  | |  | | |  |
| 20.8 | | | | 44.9 | | | 13.2 | 7.6 | | 45.3 | | |  |
| 17.5 | | | | 62.8 | | | 83.9 | 83.9 | |  | | |  |
| 20.1 | | | | 48.9 | | | 31.9 | 32.7 | | 24.3 | | |  |
| 29.3 | | | | 76.3 | | | 51.3 | 52 | | 45 | | |  |
| 14.1 | | | | 28 | | | 65.7 | 65.7 | |  | | |  |
| 15.3 | | | | 36.2 | | | 100.4 | 100.4 | |  | | |  |
| 21 | | | | 93.5 | | | 41.7 | 41.7 | |  | | |  |
| 15.4 | | | | 60 | | | 114 | 114 | |  | | |  |
| 18.2 | | | | 72.2 | | | 44.8 | 40.2 | | 68.6 | | |  |
| 14.7 | | | | 49.9 | | | 21.3 | 15.1 | | 67.8 | | |  |
| 24.7 | | | | 67.7 | | | 44 | 40.7 | | 62.7 | | |  |
| 23.2 | | | | 59 | | | 27 | 27.2 | | 21.8 | | |  |
| 29.8 | | | | 80.4 | | | 22.1 | 25.1 | | 5.3 | | |  |
| 23.5 | | | | 41.4 | | | 75.1 | 75.1 | |  | | |  |
| 18.8 | | | | 93.6 | | | 48.9 | 49.8 | | 40.7 | | |  |
| 17.9 | | | | 54.6 | | | 44.5 | 44.5 | |  | | |  |
| 18.4 | | | | 38.2 | | | 61.6 | 61.6 | |  | | |  |
| 23.9 | | | | 93.7 | | | 21.2 | 15.5 | | 38.4 | | |  |
| 29.1 | | | | 76.6 | | | 63.1 |  | |  | | |  |
| 19.4 | | | | 49.3 | | | 21.6 | 21.6 | | 21.8 | | |  |
| 21.9 | | | | 54.4 | | | 29.4 | 23.3 | | 62.3 | | |  |
| 21.1 | | | | 42.2 | | | 48.5 | 48.5 | |  | | |  |
| 43 | | | | 73 | | | 57.9 | 57.9 | |  | | |  |
| 30 | | | | 60 | | | 69 |  | |  | | |  |
| 20.9 | | | | 44.1 | | | 5.4 |  | |  | | |  |
| 16.7 | | | | 38.1 | | | 12.1 | 7.7 | | 69.6 | | |  |
| 21.6 | | | | 68.6 | | | 57.3 | 57.3 | |  | | |  |
| 22.9 | | | | 37.4 | | | 35.6 | 35.6 | |  | | |  |
| 18.2 | | | | 38.2 | | | 20.4 | 20.8 | | 12.4 | | |  |
| 21.7 | | | | 48.2 | | | 59 | 59 | |  | | |  |
| 20.4 | | | | 80.2 | | | 114.6 | 114.6 | |  | | |  |
| 17.7 | | | | 50.8 | | | 89.8 | 89.8 | |  | | |  |
|  | | | |  | | | 27.9 |  | |  | | |  |
| 18.1 | | | | 31.5 | | | 83.8 | 83.8 | |  | | |  |
| 15.1 | | | | 66.6 | | | 118.7 | 118.7 | |  | | |  |
| 18 | | | | 34.7 | | | 74.2 | 74.2 | |  | | |  |
| 19.1 | | | | 30 | | | 27.9 | 27.9 | |  | | |  |
| 28.4 | | | | 115.5 | | | 31.2 | 40.8 | | 47.7 | | |  |
| 29.3 | | | | 60.1 | | | 24.2 | 21.4 | | 49.8 | | |  |
| 19.8 | | | | 58.9 | | | 31 | 22.6 | | 45.9 | | |  |
| 14.7 | | | | 45.9 | | | 97.4 | 96.8 | | 98.7 | | |  |
| 33.1 | | | | 57.5 | | | 67.1 | 67.3 | | 64.6 | | |  |
| 18.2 | | | | 36.4 | | | 17.3 | 21.3 | | 1.2 | | |  |
| 19.2 | | | | 49.1 | | | 65.5 | 65.5 | |  | | |  |
| 17.7 | | | | 44.2 | | | 100.5 | 100.5 | |  | | |  |
| 18.7 | | | | 105.5 | | | 74 | 76.5 | | 63.9 | | |  |
| 12.7 | | | | 16.4 | | | 2.9 |  | |  | | |  |
| 16.7 | | | | 29.2 | | | 6.2 |  | |  | | |  |
|  | | | |  | | |  |  | |  | | |  |
| 23.8 | | | | 38.4 | | | 7.8 | 7.8 | |  | | |  |
| 19 | | | | 37 | | | 6 | 5.6 | | 8.5 | | |  |
| 13.8 | | | | 19.7 | | | 3.9 | 4 | | 3.9 | | |  |
| 18.7 | | | | 32.9 | | | 3.2 | 2 | | 7.3 | | |  |
| 19.2 | | | | 62.1 | | | 8.9 |  | |  | | |  |
| 14.6 | | | | 28.5 | | | 6.9 |  | | 27.7 | | |  |
| 21.5 | | | | 47.6 | | | 10.5 |  | |  | | |  |
| 22.1 | | | | 39.7 | | | 0.7 |  | |  | | |  |
| 23.1 | | | | 58.3 | | | 9.2 | 9.2 | |  | | |  |
| 23.7 | | | | 59.2 | | | 11.8 | 9.6 | | 22 | | |  |
| 20 | | | | 38.4 | | | 5.6 |  | |  | | |  |
| 30.5 | | | | 92.2 | | | 4.8 | 4.6 | | 5.7 | | |  |
| 23.5 | | | | 36.7 | | | 11.1 |  | |  | | |  |
| 12.3 | | | | 20.1 | | | 10.1 |  | |  | | |  |
| 24.7 | | | | 35.2 | | | 2.9 |  | |  | | |  |
| 31.4 | | | | 74.8 | | | 5 |  | |  | | |  |
| 16.7 | | | | 33.1 | | | 6.2 |  | |  | | |  |
| 19.4 | | | | 28.4 | | | 3 |  | |  | | |  |
| 14.8 | | | | 21.8 | | | 0.9 |  | |  | | |  |
| 13.8 | | | | 19.7 | | | 3.9 | 4 | | 3.9 | | |  |
| 20.8 | | | | 74.6 | | | 9.5 |  | |  | | |  |
| 19.8 | | | | 32.9 | | | 3.5 | 2.9 | | 6.9 | | |  |
| 18.2 | | | | 29.4 | | | 2.5 |  | |  | | |  |
| 24.3 | | | | 38.5 | | | 0.8 |  | |  | | |  |
| 17.3 | | | | 26.5 | | | 6.2 |  | |  | | |  |
| 17.4 | | | | 80.5 | | | 12.6 |  | |  | | |  |
| **minSaO2 (%)** | | | | **meanSaO2 (%)** | | | **SaO2<90% (min)** | **SaO2<90% (%)** | | **TYPE OF THERAPY** | | |  |
| 78 | | | | 93.1 | | | 27.3 | 17.7 | | BiPAP. S/T | | |  |
| 88 | | | | 94.9 | | | 0.2 | 0.2 | | autoCPAP | | |  |
| 79 | | | | 91.5 | | | 12.6 | 23.1 | | BiPAP. S/T | | |  |
| 76 | | | | 95.1 | | | 13.1 | 4.6 | | BiPAP | | |  |
| 70 | | | | 85.2 | | | 125 | 97.7 | | autoCPAP | | |  |
| 82 | | | | 91.6 | | | 7.1 | 13.2 | | autoCPAP | | |  |
| 90 | | | | 93 | | | 0.1 | 0 | | autoCPAP | | |  |
| 83 | | | | 91.6 | | | 25 | 8.1 | | BiPAP | | |  |
| 89 | | | | 94.1 | | | 0.1 | 0.1 | | autoCPAP | | |  |
| 85 | | | | 90.7 | | | 28.4 | 14.8 | | CPAP | | |  |
| 83 | | | | 91.3 | | | 3.1 | 7 | | autoCPAP | | |  |
| 77 | | | | 91 | | | 27 | 15.5 | | nt | | |  |
| 67 | | | | 87.2 | | | 21.1 | 57.3 | | BiPAP | | |  |
| 62 | | | | 87.1 | | | 283.5 | 81.7 | | BiPAP | | |  |
| 85 | | | | 92.7 | | | 0.5 | 0.5 | | autoCPAP | | |  |
| 76 | | | | 91.1 | | | 12.9 | 20.8 | | BiPAP | | |  |
| 83 | | | | 88.4 | | | 87 | 82.8 | | Oxygen therapy | | |  |
| 79 | | | | 93.9 | | | 1.7 | 1.1 | | autoCPAP | | |  |
| 74 | | | | 91 | | | 19.7 | 5.1 | | autoCPAP | | |  |
| 88 | | | | 93.6 | | | 0.5 | 0.3 | | CPAP | | |  |
| 79 | | | | 91.6 | | | 11 | 14.9 | | BiPAP | | |  |
| 79 | | | | 88 | | | 39.3 | 9.3 | | autoCPAP | | |  |
| 88 | | | | 93.3 | | | 0.7 | 0.6 | | ASV | | |  |
| 88 | | | | 94.2 | | | 0.7 | 0.7 | | autoCPAP | | |  |
| 82 | | | | 93.9 | | | 9 | 9.6 | | autoCPAP | | |  |
| 78 | | | | 91 | | | 5.8 | 3.3 | | autoCPAP | | |  |
| 85 | | | | 93.9 | | | 0.4 | 0.5 | | autoCPAP | | |  |
| 80 | | | | 94.4 | | | 8.8 | 2.4 | | nt | | |  |
| 71 | | | | 92 | | | 102.9 | 25.2 | | BiPAP. S/T | | |  |
| 84 | | | | 95.3 | | | 0 | 0 | | autoCPAP | | |  |
| 89 | | | | 92.8 | | | 0.1 | 0.1 | | CPAP | | |  |
| 82 | | | | 92.4 | | | 4.3 | 12.2 | | autoCPAP | | |  |
| 89 | | | | 91.7 | | | 0.3 | 0.1 | | autoCPAP | | |  |
| 76 | | | | 93.6 | | | 6.3 | 8.3 | | BiPAP. S/T | | |  |
| 82 | | | | 92 | | | 2.3 | 1.2 | | BiPAP. S/T | | |  |
| 81 | | | | 91.7 | | | 6.3 | 33.1 | | autoCPAP | | |  |
| 77 | | | | 91 | | |  |  | | autoCPAP | | |  |
| 73 | | | | 90.7 | | | 9.8 | 37.5 | | autoCPAP | | |  |
| 85 | | | | 92.8 | | | 10.6 | 6.6 | | autoCPAP | | |  |
| 76 | | | | 88.8 | | | 71.3 | 61.8 | | autoCPAP | | |  |
| 75 | | | | 93.6 | | | 3.1 | 2.1 | | autoCPAP | | |  |
| 86 | | | | 95.3 | | | 1.9 | 0.7 | | CPAP | | |  |
| 77 | | | | 85.4 | | | 237.8 | 95.2 | | BiPAP. Oxygen therapy | | |  |
| 78 | | | | 91.9 | | | 29.6 | 25.5 | | autoCPAP | | |  |
| 82 | | | | 88.7 | | | 51.6 | 43.2 | | BiPAP | | |  |
| 90 | | | | 96.7 | | | 0 | 0 | | autoCPAP | | |  |
| 84 | | | | 90.8 | | | 20.8 | 19.7 | | BiPAP | | |  |
| 75 | | | | 91.8 | | | 17.6 | 5.9 | | autoCPAP | | |  |
| 78 | | | | 92.8 | | | 15.9 | 14 | | autoCPAP | | |  |
| 85 | | | | 97 | | | 0.8 | 0.3 | | autoCPAP | | |  |
| 82 | | | | 92.4 | | | 2.9 | 2.1 | | autoCPAP | | |  |
| 86 | | | | 90 | | | 4.4 | 10 | | autoCPAP | | |  |
| 83 | | | | 94 | | | 0.5 | 0.2 | | autoCPAP | | |  |
| 51 | | | | 89.6 | | | 147 | 51.4 | | autoCPAP | | |  |
| 83 | | | | 89.9 | | | 68 | 38.1 | | BiPAP | | |  |
| 71 | | | | 97.2 | | | 1.7 | 0 | | nt | | |  |
| 85 | | | | 92 | | | 2.1 | 0.2 | | BiPAP. οξυγονοθεραπεία | | |  |
| 82 | | | | 93.9 | | | 6.4 | 4.4 | | autoCPAP | | |  |
| 84 | | | | 93.9 | | | 0.2 | 0.1 | | autoCPAP | | |  |
| 85 | | | | 93.9 | | | 2.9 | 2.9 | | BiPAP. S/T | | |  |
| 67 | | | | 88.5 | | | 7.6 | 38.7 | | BiPAP | | |  |
|  | | | |  | | |  |  | | nt | | |  |
| 79 | | | | 89 | | | 25.4 | 6 | | autoCPAP | | |  |
| 87 | | | | 92.8 | | | 0.5 | 2.8 | | autoCPAP | | |  |
| 62 | | | | 83.7 | | | 112.8 | 85.3 | | CPAP. Oxygen therapy | | |  |
| 90 | | | | 94.7 | | | 0 | 0 | | autoCPAP | | |  |
| 80 | | | | 91.7 | | | 1 | 1.4 | | autoCPAP | | |  |
| 79 | | | | 90.1 | | | 38.7 | 32.1 | | autoCPAP | | |  |
| 82 | | | | 93.4 | | | 10 | 3.6 | | autoCPAP | | |  |
| 71 | | | | 94.5 | | | 13.6 | 4.5 | | autoCPAP | | |  |
| 71 | | | | 88.5 | | | 220.2 | 62.9 | | BiPAP | | |  |
| 76 | | | | 92.5 | | | 52.7 | 29 | | BiPAP | | |  |
| 90 | | | | 95.5 | | |  |  | | nt | | |  |
| 85 | | | | 92.4 | | | 4.6 | 12.3 | | autoCPAP | | |  |
| 74 | | | | 87.6 | | | 37.1 | 50.7 | | autoCPAP | | |  |
| 57 | | | | 84 | | | 187.7 | 45.4 | | autoCPAP | | |  |
| 86 | | | | 92.4 | | | 1.3 | 0.4 | | nt | | |  |
| 87 | | | | 93.4 | | | 0.4 | 0.2 | | nt | | |  |
|  | | | |  | | |  |  | | nt | | |  |
| 91 | | | | 95.1 | | | 0 | 0 | | ENT | | |  |
| 91 | | | | 96 | | | 0 | 0 | | ENT | | |  |
| 84 | | | | 93 | | | 10.7 | 6 | | nt | | |  |
| 83 | | | | 96.1 | | | 0.3 | 0.2 | | nt | | |  |
| 87 | | | | 94.8 | | | 0.3 | 0.1 | | nt | | |  |
| 86 | | | | 91 | | | 0.9 | 0.2 | | nt | | |  |
| 83 | | | | 94.2 | | | 3 | 0.9 | | nt | | |  |
| 91 | | | | 95.5 | | | 0 | 0 | | nt | | |  |
| 95 | | | | 87 | | | 1.6 | 0.4 | | ENT | | |  |
| 78 | | | | 94 | | | 7 | 1.7 | | SLEEP HYGIENE | | |  |
| 86 | | | | 94.1 | | | 3.4 | 1.6 | | nt | | |  |
| 87 | | | | 92 | | | 2.6 | 1.5 | | nt | | |  |
| 83 | | | | 90.3 | | | 115.2 | 31.5 | | nt | | |  |
| 90 | | | | 94.3 | | | 1.3 | 0.1 | | nt | | |  |
| 90 | | | | 93.3 | | | 0 | 0 | | nt | | |  |
| 87 | | | | 94.5 | | | 3.9 | 2 | | nt | | |  |
| 85 | | | | 91.8 | | | 4.7 | 2 | | nt | | |  |
| 88 | | | | 95.1 | | | 0.1 | 0.1 | | nt | | |  |
| 89 | | | | 91.7 | | | 2.4 | 1.3 | | nt | | |  |
| 84 | | | | 93 | | | 10.7 | 6 | | nt | | |  |
| 84 | | | | 93.2 | | | 1.1 | 0.6 | | nt | | |  |
| 87 | | | | 92.6 | | | 2.7 | 1.6 | | nt | | |  |
| 78 | | | | 88.1 | | | 251.7 | 87.9 | | Oxygen therapy | | |  |
| 94 | | | | 95 | | | 0 | 0 | | nt | | |  |
| 90 | | | | 94.3 | | | 0 | 0 | | nt | | |  |
| 85 | | | | 94.3 | | | 0.6 | 0.3 | | nt | | |  |
